# Magnesium, Oxidative Stress, Inflammation, and Cardiovascular Disease

**DOI:** 10.3390/antiox9100907

**Published:** 2020-09-23

**Authors:** Man Liu, Samuel C. Dudley

**Affiliations:** Division of Cardiology, Department of Medicine, the Lillehei Heart Institute, University of Minnesota at Twin Cities, Minneapolis, MN 55455, USA

**Keywords:** antioxidant, inflammation, mitochondria, arrhythmias, heart failure with preserved ejection fraction, heart failure with reduced ejection fraction

## Abstract

Hypomagnesemia is commonly observed in heart failure, diabetes mellitus, hypertension, and cardiovascular diseases. Low serum magnesium (Mg) is a predictor for cardiovascular and all-cause mortality and treating Mg deficiency may help prevent cardiovascular disease. In this review, we discuss the possible mechanisms by which Mg deficiency plays detrimental roles in cardiovascular diseases and review the results of clinical trials of Mg supplementation for heart failure, arrhythmias and other cardiovascular diseases.

## 1. Introduction

Cardiovascular disease (CVD) refers to a number of conditions such as heart failure (HF), arrhythmia, atherosclerosis, and stroke. Two types of HF can occur when the heart fails to pump enough blood to support other organs in the body. One is systolic HF as a result of poor contractile function, also known as HF with reduced ejection fraction (HFrEF). The other is diastolic HF as a result of reduced heart filling in diastole, also known as HF with preserved ejection fraction (HFpEF). Recently, our group has reported that magnesium (Mg) supplementation demonstrates significant protective effects on cardiac diastolic function, providing a new possible therapy for HFpEF [1]. Therefore, review of the effects of Mg on CVD seems timely.

CVDs are the number one cause of death globally. About 650,000 Americans die from CVD each year. In the United States, about 6.5 million adults have HF, compared with ~5.7 million between 2009–2012 [2]. HF caused one in eight deaths in 2018 [3], in spite of the remarkable advances in therapeutic treatments and prevention. Certain medical conditions increase risk for HF, such as diabetes mellitus (DM), obesity, and hypertension. Primary prevention of HF starts with a healthy lifestyle by following the Life’s Simple 7 goals (diet, smoking, body mass index, physical activity, cholesterol, glucose, and blood pressure) [2]. Treatment of systolic HF can involve medicines, mechanical support, arrhythmia prevention, and transplantation. There are no specific therapies for diastolic HF.

Hypomagnesemia is commonly observed in HF [4,5,6,7,8,9,10], DM [1,11], CVD [6,12,13,14], hypertension [15,16], and stroke [17,18], and the presence of hypomagnesemia in these conditions has been nicely reviewed [6,7,8,19,20]. Dietary Mg intake is inversely correlated with the occurrence of metabolic diseases [21,22], such as DM (type 1, 2 and 3) [23,24,25,26] and hypertension [27,28]. Low serum Mg is a predictor for cardiovascular and all-cause mortality [13], and hypomagnesemia is associated with unstable cardiac electrical repolarization and contributes to sudden death in HF [29].

Increased consumption of processed food, filtered/deionized drinking water, and crops grown in Mg-deficient soil has led to a significant decline in Mg intake [30,31,32]. The majority of North Americans consume 185–235 mg/day Mg, compared with 450–485 mg/day in ~1900 [33]. Studies have reported that ~50% of the US population, especially the elderly, consume less than the estimated average requirement for Mg [33,34,35] and ~23% of US adults have hypomagnesemia (serum Mg <0.7 mM or 1.7 mg/dL) [36,37]. Dietary Mg intake <2.3 mg/kg body weight is related to increased risk of HF hospitalization in black adults [38]. Moreover, chronic diseases and medications such as diuretics, thiazides, cytotoxic drugs, digoxin, aminoglycosides, and steroids can further increase Mg loss, decrease Mg absorption, and cause hypomagnesemia. 

Mg supplementation has shown significant therapeutic effects in HF and CVD (see reviews [4,6,12,14,39,40]). It has also been shown to improve arrhythmias including torsades de pointes (TdP, polymorphic ventricular tachycardia with marked QT prolongation on the electrocardiogram) [41,42], atrial fibrillation (AF) [43,44,45], ventricular arrhythmias (VA) [46,47], and arrhythmias in acute myocardial infarction (MI) [48].

Mg is the fourth most abundant mineral and the second most abundant intracellular divalent cation. It acts as a cofactor for more than 300 metabolic reactions, such as ATP production [49,50,51], protein synthesis, DNA/RNA synthesis [52], and mitochondrial function maintenance [53,54]. As a natural calcium (Ca) antagonist, Mg also participates in the regulation of Ca homeostasis. Mg has been reported to play critical roles in heart rhythm [55,56,57,58], muscle contraction [59,60,61], blood pressure [15,16,62], insulin/glucose metabolism [63,64], and bone integrity [65,66,67]. In this review, we will focus on the effects of Mg on CVD. First, we will discuss the mechanisms of how Mg deficiency affects metabolism including Mg deficiency-induced oxidative stress, inflammation, and insulin resistance. Then, we will review clinical and animal studies on how Mg supplementation has been used to improve HF, arrhythmias, metabolic syndromes, and vascular diseases. In the end, we will discuss the methods of Mg treatment including formulations, routes of delivery, dosing, and duration in different diseases. The limitations of Mg as a treatment will also be discussed.

## 2. Mg Deficiency Induces Metabolic Derangements

Normal serum Mg concentration is between 0.7–1.0 mM (1.7–2.4 mg/dL) [68,69]. Hypomagnesemia is defined as serum Mg < 0.7 mM. Mild to moderate hypomagnesemia occurs when serum Mg is between 0.5–0.69 mM (1.20–1.88 mg/dL) and severe hypomagnesemia is a serum Mg < 0.5 mM. For cellular Mg, about half of the total Mg is bound to nucleotide triphosphates, primarily ATP (MgATP), and ~20% is in the cytoplasm and the lumen of organelles. In cardiomyocytes, free ionized intracellular Mg (Mg_i_) concentration ([Mg]_i_) is tightly maintained in the range of 0.8–1.0 mM [70,71]. 

Several Mg transporters and channels have been identified in the heart and regulate Mg influx and efflux across the plasma membrane, including the transient receptor potential melastatin 7 channel (TRPM7, mainly for Mg influx), solute carrier family 41 A1 (SLC41A1, for Mg efflux), Mg transporter 1 (MagT1, for Mg influx), and the ancient conserved domain protein 2 (ACDP2, or cyclin and CBS domain divalent metal cation transport mediator 2: CNNM2, for Mg influx) [72,73,74]. Mitochondrial RNA splicing 2 protein (MRS2, for Mg influx into mitochondria) [75] and solute carrier family 41 A3 (SLC41A3) (for Mg efflux from mitochondria) [76] appear to transport Mg across the mitochondrial membranes. Dysregulation of these Mg transporters and channels is caused by and contributes to disturbed Mg homeostasis [72,77,78]. For example, gene upregulation of SLC41A1 and MagT1 have been reported with Mg deficiency [72,79,80]. Under hypomagnesemia, low serum (i.e., extracellular) Mg levels can activate Mg transporters such as TRPM7 and SLC41A1 to induce Mg efflux from cells to increase serum Mg levels. This would be expected to decrease [Mg]_i_ and affect Mg/MgATP-dependent cellular signaling and functions. A decreased [Mg]_i_ could trigger Mg stores such as mitochondria [51,81] to release Mg through SLC41A3 [76]. Decreased mitochondrial Mg levels ([Mg]_m_) could affect further Mg/MgATP-associated mitochondrial signaling and functions, which may explain the mitochondrial reactive oxygen species (ROS) overproduction and decreased ATP levels observed in Mg deficient mice [1,82].

CVD patients often have hypomagnesemia, resulting from low Mg intake and increased Mg loss. In a study of congestive HF, 38% of patients have hypomagnesemia, and 72% of patients have excessive Mg loss [10]. This could be attributed to electrolyte disturbances induced by the excessive activation of the renin-angiotensin-aldosterone system (RAAS) [5,83,84,85] and by long-term use of medications such as diuretics and digoxin in chronic HF [9,86,87,88]. Hypomagnesemia may increase the risk of CVD by suppressing mitochondrial function with increased oxidative stress and decreased energy production [1,50,89,90], modulating cardiac ion channels to cause myocardium electrical remodeling [8,47,56], and disturbing Ca homeostasis and cardiac contraction [1,8,49,82,91]. Mg deficiency also induces sustained inflammation [35,92,93,94,95] and interrupts insulin signaling [26,64,96,97], both of which contribute to the development and progression of CVD. In this section, we will discuss possible mechanisms of Mg deficiency-associated CVD, including Mg deficiency-induced oxidative stress, inflammation, and insulin resistance.

### 2.1. Mg Deficiency and Oxidative Stress

Oxidative stress is common in CVD [98,99,100,101]. Mg deficiency has been associated with oxidative stress in HF, DM, obesity, and hypertension [1,4,5,9,86,102,103] (for reviews [77,104,105]). Obesity, which is a strong risk factor for CVD, is linked with decreased serum Mg and increased malondialdehyde, a marker for oxidative stress resulting from lipid peroxidation [106,107]. ROS in the heart are mainly produced by mitochondria, nicotinamide adenine dinucleotide phosphate (NADPH) oxidases, uncoupled nitric oxide synthase (NOS), and xanthine oxidase. Mitochondria occupy ~30% of the cardiomyocyte volume and are not only the major site for energy and ROS production in the heart but also are major Mg stores (with three- to five-fold higher concentrations of [Mg]_m_ compared with [Mg]_i_) [75,108]. Recently, our group has reported that Mg deficiency in diabetic mice increases mitochondrial oxidative stress and contributes to cardiac diastolic dysfunction [1]. Both mitoTEMPO (a mitochondria-targeted ROS scavenger, (2-(2,2,6,6-Tetramethylpiperidin-1-oxyl-4-ylamino)-2-oxoethyl)triphenylphosphonium chloride) treatment [109] and Mg supplementation [1] reverse diastolic function by reducing the oxidative stress and subsequent *S*-glutathionylation of cardiac myosin binding protein C. In a low-Mg diet-induced Mg deficient mouse model, mitochondrial oxidative stress is also detected and contributes to cardiac diastolic dysfunction [82]. Both mitoTEMPO treatment and Mg repletion can suppress mitochondrial ROS overproduction and reverse diastolic dysfunction [82]. Therefore, Mg can act as a mitochondrial antioxidant.

**Figure 1:**
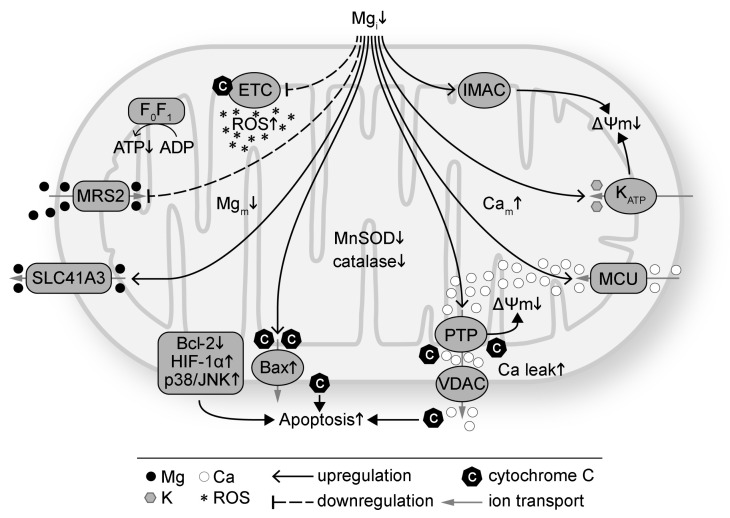
Intracellular magnesium (Mg) deficiency (Mg_i_↓) induces mitochondrial oxidative stress and dysfunction. Mg deficiency downregulates the electron transport chain (ETC) and increases reactive oxygen species (ROS) production. Mg deficiency also suppresses the antioxidant defense system with decreased protein levels such as manganese SOD (MnSOD) and catalase. Mg deficiency downregulates ATP synthase (F0F1) and decreases ATP production. Intracellular Mg deficiency inhibits Mg transport into mitochondria via mitochondrial RNA splicing 2 protein (MRS2) and promotes mitochondrial Mg (Mg_m_) efflux via SLC41A3. Mg deficiency promotes apoptosis by increasing cytochrome C release via Bax and voltage dependent anion channel (VDAC), decreasing anti-apoptotic proteins such as the Bcl-2 family, and increasing pro-apoptotic proteins such as HIF-1α and p38/JNK. Mg deficiency induces mitochondrial membrane (ΔΨ_m_) depolarization by increasing the permeability of mitochondrial permeability transition pore (PTP), ATP-sensitive K channel (K_ATP_), and inner membrane anion channel (IMAC). Mg deficiency increases mitochondrial Ca (Ca_m_) via the mitochondrial Ca uniporter (MCU). Ca leak from mitochondria via VDAC is also increased with Mg deficiency.

Presented in Figure 1 are the effects of Mg deficiency on mitochondria. Intracellular Mg deficiency has been shown to disrupt mitochondrial function by altering coupled respiration [50,89,90], increasing mitochondrial ROS production [1,82,110], suppressing the antioxidant defense system (such as superoxide dismutase (SOD), catalase, glutathione, and Parkinsonism associated deglycase PARK7/DJ-1) [105,111,112,113,114], inducing mitochondrial Ca overload via the mitochondrial Ca uniporter [1,54,115], attenuating pro-survival signaling [116,117,118], and promoting opening of mitochondrial ATP-sensitive potassium (K) channel [119], inner membrane anion channel [120] and mitochondrial permeability transition pore (PTP) [121]. The effects result in depolarization of the mitochondrial membrane potential (ΔΨ_m_) [54]. Mg repletion, on the other hand, has been found to improve mitochondrial function by suppressing mitochondrial ROS overproduction [1,82], inhibiting mitochondrial PTP opening and cytochrome C release [122,123,124], preserving ΔΨ_m_ [53,125], diminishing mitochondrial Ca accumulation [1,49,126,127], enhancing protein expression of the anti-apoptotic B-cell lymphoma 2 (Bcl-2) family and concomitantly decreasing pro-apoptotic protein expression such as Bcl-2-associated X protein (Bax) [118,125], decreasing apoptosis by suppressing activation of hypoxia-inducible factor 1α (HIF-1α) and p38 mitogen activated protein kinase/c-Jun N-terminal kinase (p38/JNK) signaling [125], and downregulating autophagy [127].

**Figure 2:**
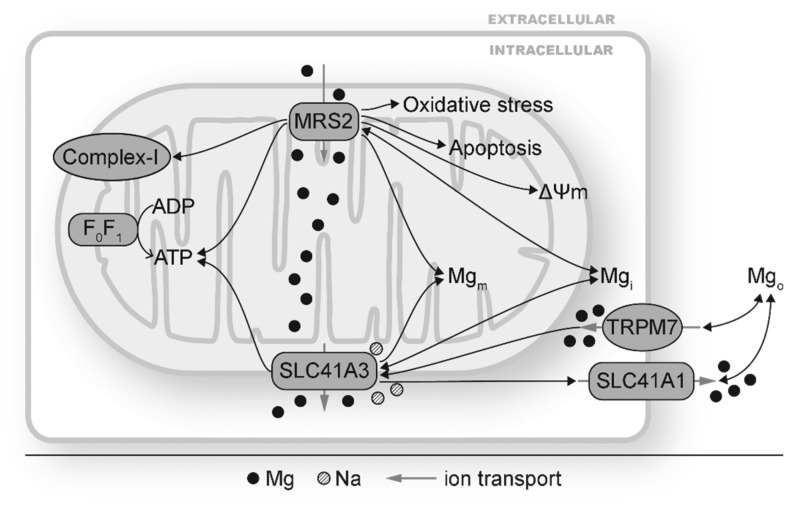
Mitochondrial Mg transporters MRS2 and SLC41A3 mediate mitochondrial and cellular changes by modulating mitochondrial Mg homeostasis. MRS2 and SLC41A3 carry out mitochondrial Mg (Mg_m_) influx and efflux, respectively. Changes in MRS2 have shown to alter Mg_m_ and affect complex I, ATP production, mitochondrial membrane depolarization (ΔΨ_m_), intracellular Mg (Mg_i_), and cellular oxidative stress and apoptosis. Changes in SLC41A3 has been reported under hypomagnesemia and TRPM7 depletion. SLC41A3 impacts ATP production, expression of other Mg transporters such SLC41A1. The double-headed black arrows indicate that changes in Mg transporters can result in and from disturbed Mg homeostasis.

There are two identified mitochondrial Mg transporters, MRS2 and SLC41A3 (Figure 1 and Figure 2). MRS2 mainly transports Mg into mitochondria, although Mg efflux via MRS2 is also observed when ΔΨ_m_ is depolarized [75]. MRS2 knockdown has been reported to reduce Mg uptake capacity, decrease [Mg]m, induce loss of complex-I of the electron transport chain, reduce cellular and nuclear ATP levels, depolarize ΔΨ_m_, make cells vulnerable to oxidative stress and apoptotic stimulation, and increase cell death (Figure 2) [51,128]. MRS2 upregulation, on the other hand, exhibits protective effects against apoptosis. Overexpression of MRS2 leads to increased total cellular Mg levels and enhanced resistance to apoptotic inducing drugs [129], probably by inhibiting Bax-induced cytochrome C release from mitochondria [118]. These results suggest the vital roles of MRS2 to maintain [Mg]_m_ and mitochondrial function.

SLC41A3 is highly expressed in the heart, central nervous system, small intestine, and lung and is responsible for Mg efflux from mitochondria [76,130]. Its exact location on either inner or outer membrane of mitochondria has not been determined yet. In Figure 2, SLC41A3 is depicted on the outer mitochondrial membrane for convenience. Under Mg deficiency, the mRNA and protein levels of SLC41A3 are increased, probably as a compensation so that the mitochondria can release Mg to cope with Mg deficiency [131]. SLC41A3 overexpression leads to significantly increased Mg efflux from mitochondrial in a sodium (Na) dependent and temperature sensitive manner [76]. Cells with SLC41A3 overexpression demonstrate lower cellular ATP levels, suggesting that a decreased [Mg]_m_ suppresses mitochondrial ATP production [76]. SLC41A3 knockout in mice results in hypomagnesemia under a normal diet, indicating that mitochondria play important roles in Mg homeostasis. Mechanisms for hypomagnesemia include increased intestinal mRNA expression of TRPM6, TRPM7, and SLC41A1 and therefore increased Mg extrusion [130]. Under Mg deficiency, the electro-chemical balance of Mg across the mitochondrial membranes will be altered. MRS2 and SLC41A3 can respond to this change by decreasing mitochondrial Mg influx and increasing mitochondrial Mg efflux, respectively. This would increase [Mg]_i_ and decrease [Mg]_m_. Fluctuation of [Mg]_m_ could lead to changes in Mg-dependent signaling cascades to modulate the mitochondrial electron transport chain, mitochondrial ATP and ROS production, and other mitochondrial functions.

Aside from the association between Mg deficiency and mitochondrial oxidative stress, studies have reported correlations between Mg deficiency and other ROS sources. Mg deficiency is found to interfere with nitric oxide (NO) release from coronary endothelium [132]. Mg deficiency results in activation of endothelial and neuronal nitric oxide synthase as well as reduction of serum Mg and tissue glutathione in all chambers of the heart [111]. Mg deficiency also causes an increased activity in NADPH oxidase induced by decreased ATP [133].

### 2.2. Mg Deficiency and Inflammation

Mg deficiency has been linked to inflammation for decades [66,94,134,135,136,137], and this association has been reviewed many times [35,81,92,138,139]. Reduced Mg levels result in activation of inflammation, while elevated Mg levels suppress the inflammatory response. Table 1 lists some inflammatory biomarkers that have been reported to be elevated under Mg deficiency. For example, Mg levels in HF are inversely related to inflammatory markers C-reactive protein (CRP) and tumor necrosis factor-α (TNF-α) [81,140]. Decreased plasma Mg is associated with increased monocyte chemoattractant protein-1 (MCP-1) [141]. In animal studies, low-Mg diet increases interleukin-1 (IL-1), IL-6, TNF-α, IL-8, and MCP-1 [66,134,136,142,143,144]. Moderate Mg deficiency (~50% of requirement) is associated with increased TNF-α, oxidative stress, and increased risk to develop CVD [142,143]. Protein kinase C (PKC) activation induced by Mg deficiency seems to contribute to the synthesis and release of cytokines [145]. 

**Figure 3:**
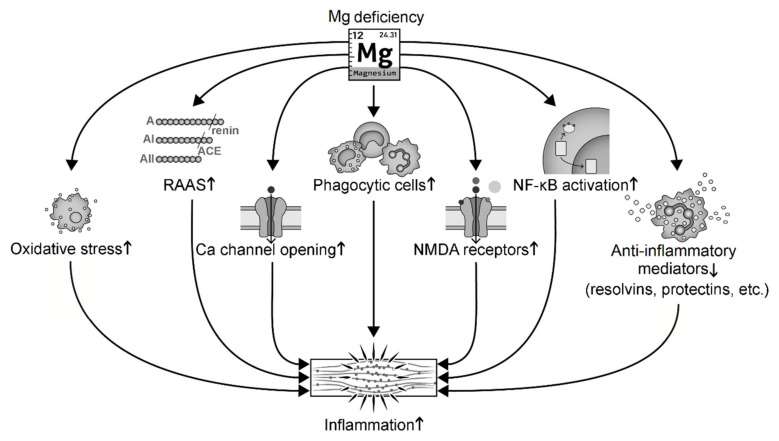
Mg deficiency induces inflammation through several signaling pathways. Abbreviation: NMDA, N-methyl-D-aspartate; RAAS, the renin-angiotensin-aldosterone system.

Hypomagnesemia activates systemic inflammation in at least seven ways (Figure 3): (i) induction of cellular oxidative stress that promotes inflammation [92,151,152]; (ii) activation of the RAAS that leads to inflammation [84,92,153]; (iii) relief of Mg inhibition on Ca channel opening that induces abnormal Ca handling [151,154,155]; (iv) activation of phagocytic cells [134,154,156,157,158]; (v) relief of Mg antagonism on the N-methyl-D-aspartate (NMDA) that leads to over-activated NMDA receptors and release of substance P [95,138,151]; (vi) activation of nuclear factor-κB (NF-κB) signaling and upregulation of transcription of cytokines and pro-inflammatory genes such as IL-1α [136,159,160,161]; and (vii) reduction of anti-inflammatory mediators such as NO, lipoxins, resolvins, and protectins [103,162]. For instance, low-Mg diet significantly increases macrophage-derived cytokines (IL-1, IL-6, and TNF-α) in hamsters and rats [134]. A few days of experimental Mg deficiency in rats induces activation of leukocytes and macrophages and increases release of inflammatory cytokines [93]. Low Mg increases the oxidative activity of neutrophils, while high Mg significantly decreases oxyradical production in rats and human polymorphonuclear cells [163,164]. Phagocyte priming has been proposed as a mechanism of Mg deficiency-induced immunoinflammatory processes [156,158].

Mg transporters TRPM7 and MagT1 have been shown to affect and be affected by inflammation. TRPM7 is highly expressed in leukocytes and many organs such as the heart and kidney. It is a rare channel-enzyme with a channel domain to transport multiple cations (including Mg and Ca) and a kinase domain to phosphorylate serine/threonine [165,166]. TRPM7 acts as a key transporter to regulate Mg homeostasis by mediating Mg influx [166,167,168]. Its channel function is inhibited by physiological [Mg]_i_ and [Ca]_i_ but activated under Mg deficiency [166]. Its kinase function is elevated by Mg [169,170]. Transgenic mice with heterozygous deletion of TRPM7 kinase domain (TRPM7^+/Δkinase^) have cardiac hypertrophy, inflammation, fibrosis, decreased cardiac [Mg]_i_, elevated expression of cytokines (IL-6, -10, -12, and TNF-α), increased cardiac collagen and fibronectin content, and increased infiltration of macrophages [171]. Inhibition of TRPM7 can abolish IL-4-induced macrophage proliferation and prevent macrophage polarization towards the anti-inflammatory M2 phenotype [172]. 

MagT1 conducts Mg influx and plays a key role in T cell-activated immune response [155,173]. MagT1 deficiency leads to the absence of a T cell antigen receptor-stimulated Mg flux and attenuation of T cell activation [155,173]. Matsuda-Lennikov et al. have reported that MagT1 mediates N-linked glycosylation and expression of immune-response genes in T cells [174]. 

### 2.3. Mg Deficiency and Insulin Resistance

Mg deficiency, insulin resistance, and oxidative stress often co-exist in patients with diabetes, obesity, and hypertension [64,175]. Mg is a second messenger for insulin signaling [20,155,176]. It affects glucose transportation across the membrane [177,178] and insulin secretion and release [179,180]. Mg can also affect insulin signaling through its regulation of redox balance and enzyme/kinase activities [1,96,181,182,183].

**Figure 4:**
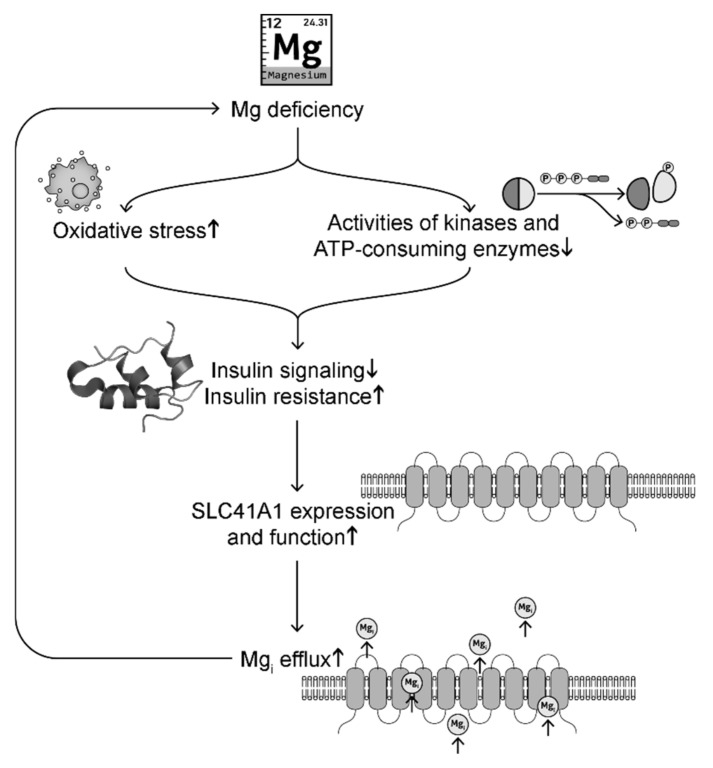
A possible signaling pathway linking intracellular Mg deficiency and insulin resistance in a positive feedback loop.

Oxidative stress induced by Mg deficiency is a major cause of disrupted insulin signaling [181]. Animal studies show that rats and mice fed with low Mg diet exhibit increased plasma glucose levels, insulin resistance, and oxidative stress [96] (and our unpublished results). Decreased serum Mg and increased oxidative stress are observed in diabetic mice, while Mg supplementation decreases blood glucose levels and improves glucose tolerance and mitochondrial oxidative stress [1]. Vitamin E, an antioxidant, has been shown to increase [Mg]_i_ levels and insulin sensitivity [184]. On the other hand, Mg, being a cofactor for adenine nucleotides, is critical for the activity of ATP-consuming enzymes and kinases (such as hexokinase, phosphofructokinase, aldolase, phosphoglycerate kinase, pyruvate kinases, protein kinase B also known as Akt2, and insulin-related substrate-1) that regulate glycolysis [64,185,186,187] and insulin action [182,183] (Figure 4). For instance, Mg supplementation upregulates Akt2 and insulin-related substrate-1 to decrease blood glucose levels and improve glucose tolerance [183]. In summary, Mg deficiency can cause downregulation of insulin signaling and elevated insulin resistance.

For decades, insulin has been reported to regulate Mg levels [63,176,188]. SLC41A1 transports Mg out of the cells. Insulin signaling has been shown to inhibit SLC41A1 expression and function [176,188]. Under insulin resistance, there is less inhibition of SLC41A1, which leads to elevated cytoplasmic Mg efflux, causing intracellular Mg deficiency [189]. Increased SLC41A1 has been observed in Mg deficiency [72,79]. Mg deficiency can in turn exacerbate oxidative stress and downregulation of enzymes and kinases involved in glycolysis and insulin signaling, forming a positive feedback. This possible signaling pathway is summarized in Figure 4. These signaling changes may explain why Mg deficiency is often observed in patients with DM [23,24,25,26]. Mg supplementation and insulin both have protective effects for [Mg]_i_ levels by inhibition of SLC41A1 expression [188,189].

## 3. Mg Supplementation as a Therapeutic Treatment for Cardiomyopathy

Chronic Mg deficiency contributes to the development and progression of HF, hypertension, atherosclerotic vascular disease, and metabolic diseases, while acute Mg deficiency has been shown to be associated with cardiac arrhythmia and neuromuscular hyper-excitability (such as pre-eclampsia and epilepsy) [190]. Recently, our group found that Mg deficiency independently induced systolic and diastolic dysfunction in mice with low Mg diet [82]. The underlined mechanisms include Mg deficiency-induced mitochondrial dysfunction, oxidative stress, oxidation of cardiac myosin binding protein C, dysregulation of Ca handling proteins such as the sarcoplasmic reticulum (SR) Ca-ATPase (SERCA) and the SR Ca release channel (i.e., the ryanodine receptor 2 (RyR2)), and suppression of Ca transient. Mg repletion reversed all the detrimental changes and restored heart function. 

**Figure 5:**
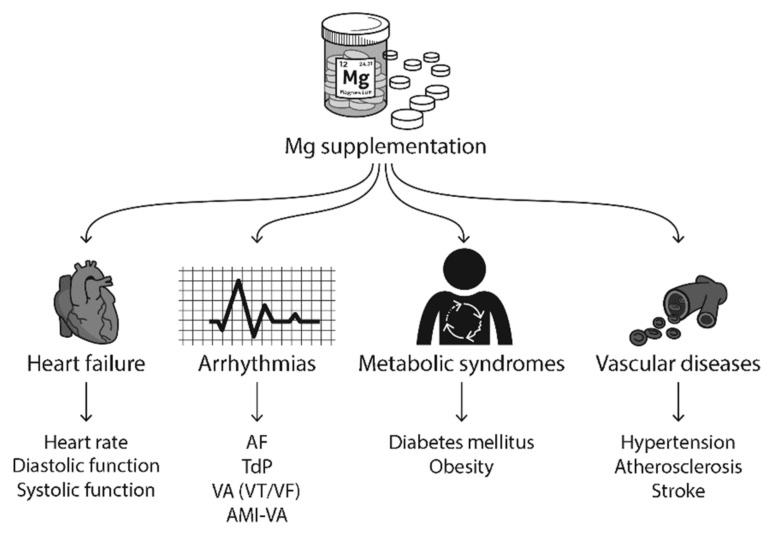
Mg supplementation has been reported to improve heart failure (HF), arrhythmias, metabolic syndromes, vascular diseases, and neuronal diseases in clinical studies. Abbreviations: AF, atrial fibrillation; TdP, torsades de pointes; VA, ventricular arrhythmias; VT, ventricular tachycardia; VF, ventricular fibrillation; AMI, acute myocardial infarction.

In this section, we will review the published clinical and experimental studies on how Mg supplementation improves HF, arrhythmias, metabolic syndromes, and vascular diseases (Figure 5).

### 3.1. Mg Supplementation Improves HF

In HF patients, hypomagnesemia is frequently observed (with a reported 7%–38% range), together with other electrolyte abnormalities such as hypocalcemia [5,9]. Maintaining a normal Mg level with Mg supplementation plays a protective role on HF survival and on all-cause mortality [191]. Gottlieb et al. reported that HF patients with low Mg levels have a two-year survival rate of 42% vs. 61% for patients with normal Mg levels [39]. Mg deficiency-induced oxidative stress and inflammation contribute to the development and progression of HF [5,81,103,142], while Mg supplementation suppresses oxidative stress and inflammation and plays protective roles in HF [1,82,152].

Benefits of Mg supplementation have been reported in many studies on treating HF patients with improvements in arrhythmias, diastolic and systolic function, inflammation, and myocardial infarction rate. For example, intravenous (IV) Mg sulfate (MgSO_4_) treatment reduced left ventricular failure by 25% compared with saline placebo in the 2nd Leicester Intravenous Mg Intervention (LIMIT-2) trial [192,193]. A follow-up study of LIMIT-2 showed that Mg treatment significantly decreased the mortality rate from ischemic heart disease by 21% and all-cause mortality rate by 16% [193]. New York Heart Association (NYHA) class II-III HF patients with MgSO_4_ infusion (40 mmol over 24 h) showed significantly reduced QT variability, and the change in QT variability was correlated inversely with patient serum Mg levels [194]. Oral Mg citrate (300 mg/day for five weeks) appears to decrease heart rate variability in HF patients, accompanied by an increased serum Mg level [57]. Oral Mg chloride (MgCl_2_) treatment (380 mg/day for six weeks) [195] or IV MgSO_4_ infusion (one dose, 0.1 mmol over 1 h or 8 g in 250 mL of 5% glucose over 12 h) [10,196] on patients with congestive HF significantly reduced the frequency of ventricular arrhythmias in HF patients and increased serum Mg levels. This indicates an acute effect of IV infusion on improving the symptoms of patients with congestive HF, while oral Mg administration may need longer treatment. One of the longest Mg treatments reported is oral Mg orotate for one year in patients with severe HF, showing significantly higher survival rate and improved clinical symptoms when compared with placebo [197]. In animal studies, our group has reported that MgSO_4_ supplementation in drinking water improves diabetes-associated diastolic dysfunction in six weeks [1], and that Mg repletion reverses low-Mg diet induced HFpEF and HFrEF [82]. 

### 3.2. Mg Supplementation Shows Protective Effects against Arrhythmias

Mg has been used to treat different types of arrhythmias for decades, such as AF [43,44,45,198,199], TdP [41,42,200], and VA [46,47,199] (for reviews [6,201,202,203,204]). Mg is also frequently used in patients undergoing cardiac and pulmonary surgery when lethal arrhythmias often occur [44,199,204,205,206]. For example, a meta-analysis of randomized controlled trials on 2069 patients demonstrates that prophylactic Mg administration (IV MgSO_4_ or MgCl_2_) reduces the risk of supraventricular tachycardia and VA after cardiac surgery [205].

Long-term mild and moderate hypomagnesemia is associated with AF [45]. A pilot trial supports oral Mg oxide supplementation for AF prevention [47]. IV MgSO_4_ decreased the heart rate and converted to sinus rhythm patients with rapid AF [207]. Higher incidence of postoperative AF is observed in patients with lower plasma Mg levels [208]. IV Mg prevents AF after cardiac and thoracic surgery [199,204,205,209] and non-cardiac thoracic surgery [198]. IV Mg also helps control the ventricular rate in patients with AF as an adjunct to digoxin [203].

TdP is most commonly caused by medications such as QT prolonging drugs [42]. Other causes for TdP include congenital long QT syndrome, electrolyte imbalance (hypomagnesemia and/or hypokalemia), bradycardia, hypothyroidism, and cardiac diseases [210]. IV Mg has been effective in treating TdP patients and is recommended as the initial therapy of choice to treat TdP, regardless of serum Mg levels [41,201,210,211]. In a study of six patients with TdP but normal Mg levels [41], IV MgSO_4_ infusion (50 mg/min for 2 h) eliminated the arrhythmia within 20–30 min in all cases. A single bolus of IV MgSO_4_ (2 g) has been shown to abolish TdP within 1–5 min in nine of 12 TdP patients [200]. In studies of canine TdP models, Mg rapidly prevents triggered ventricular tachycardia (VT) and eliminates TdP, probably by inhibiting early afterdepolarizations, a cellular arrhythmic mechanism [212,213].

Mg has been shown to alleviate other VAs in many studies [46,47]. Oral MgCl_2_ intake decreased the occurrence of ventricular premature complexes, couplets and non-sustained VT in HF patients [195]. Oral Mg intake together with potassium exhibits antiarrhythmic effects on patients with frequent VA [46]. IV MgSO_4_ administration reduced the numbers and lasting time of ventricular ectopic beats, couplets, and episodes of nonsustained VT [10,196]. A meta-analysis on 22 studies with over 6000 patients shows that the rates of VA (VT and ventricular fibrillation (VF)) and the incidence of supraventricular tachycardia are significantly lower in patients receiving IV MgSO_4_ (11.9% and 10.4%, respectively) than placebo (24.2% and 23.9%, respectively) [91]. Intraoperative Mg treatment is associated with reduced occurrence of postoperative arrhythmias including VT, junctional ectopic tachycardia, and atrioventricular block [199].

Mg shows protective effects on arrhythmias in MI. Low serum Mg is associated with increased risk and mortality of acute MI [191,214], and Mg deficiency, in turn, aggravates MI by inducing mitochondrial dysfunction and increasing oxidative stress-induced ischemic injury [96,110,215,216]. In Mg-treated patients, there is ~20% reduction in infarct size [217], ~24%–50% decreased mortality [20,48,192,214,218,219], decreased rates of arrhythmias after infarction [48,217,218,219,220], increased ejection fraction [221], and improved myocardial contractile function [193]. For example, in a meta-analysis with eight clinical trials, IV Mg treatment of 930 acute MI patients showed a 49% reduction in VT/VF, a 58% reduction of incidence of cardiac arrest, and a 54% reduction in mortality [48]. The mechanisms underlying the protective effects of Mg have been studied in animal MI models. Mg reduces Ca overload [49,53,216,222,223], improves cellular ATP production [49], reduces myocardial oxygen consumption [49,53], attenuates catecholamine-induced elevated oxygen demand and myocardial necrosis [216,223], and protects the post-ischemic myocardium from oxidative damage [53,224]. Mg has been proposed as an adjunct therapy option in selected cases of high-risk acute MI patients, such as elderly patients, those with left ventricular dysfunction and chronic HF, and patients not suitable for reperfusion therapy [12].

The detrimental effects of Mg deficiency in arrhythmias and the protective roles of Mg supplementation have been thought traditionally to arise from Mg regulation of multiple cardiac ion channels, transporters and ionic exchangers that are responsible for the cardiac action potential, including the cardiac sodium channel [225], L-type Ca channel [58,226,227,228], T-type Ca channel [229], Na/Ca exchanger (NCX) [230,231], and K channels [232,233,234,235,236,237], such as the human ether-à-go-go-related K channel, the slowly inactivating K channel, and inward-rectifier K channels (for reviews [6,56,91,238]). Several mechanisms have been proposed for how Mg plays its roles, such as acting as a channel pore blocker, binding directly on channels, altering membrane surface potential, modulating ATP-consuming kinases and enzymes, and mediating allosteric effects on channels (see reviews [6,56,201,238]). For the cardiac sodium channel, Mg deficiency leads to a downregulation of channel function [225], which can lead to increased risks of arrhythmias by virtue of decreased conduction velocity. Mg has been proposed as a Ca antagonist for Ca channels including the L-type Ca channel, the T-type Ca channel, the NCX, and the SR RyR2. Mg_i_ has at least two effects on L type Ca currents: inhibition of channel open probability and on channel activity under protein kinase A phosphorylation [58,226,227,228]. Under low [Mg]_i_, the NCX current is significantly increased, compared to under physiological concentration [230,231], which leads increased incidence of triggered arrhythmia [231]. Physiological concentrations of [Mg]_i_ mainly inhibit Ca-induced Ca release from RyR2 [239] by competing with Ca [240,241]. Under Mg deficiency, increased Ca leak from RyR2 disturbs Ca homeostasis and increases the occurrence of early and delayed afterdepolarizations. Mg_i_ at physiological concentrations mainly plays an inhibitory role on cardiac K channels by interfering with the passage of K ions and reducing the channel open probability [242]. Under Mg deficiency, K currents are increased [232,233,234,235,236,237], which can lead to arrhythmias [243]. Under Mg deficiency, the comprehensive effects of Mg on multiple cardiac ion channels, transporters, and ion exchangers lead to prolongation of the action potential duration, increasing the risk of triggered electrical activity, while Mg supplementation reverses these effects and alleviates arrhythmias. 

The protective effects of Mg supplementation may also result from the suppression of Mg on oxidative stress and inflammation, which are common in patients with CVD such as HF [244,245] and AF [246,247,248,249,250]. Oxidative stress can induce fibrosis and electrical remodeling [246,247,248,251]. Our group has reported that Mg deficiency induces oxidative stress [1,82], which promotes arrhythmias [252] and downregulations on multiple cardiac ion channels and transporters including cardiac sodium channel [251,253,254,255], Ca channels [255,256,257,258], NCX [259], RyR2 [260], and K channels [255,256,261,262,263]. Mg supplementation can inhibit ROS overproduction [1,82] and reverse oxidative stress-induced channel changes. Mg deficiency has also shown to activate PKC [145], which can downregulate the cardiac sodium channel [251,253]. Inflammation that can be induced by Mg deficiency [81] is also associated with increased arrhythmic risks [264,265] and the suppression of Mg supplementation on inflammation [146,161] should have protective effects. 

### 3.3. Mg Treatment of Other Cardiovascular Diseases

Mg regulation of insulin signaling speaks to its importance in metabolic diseases such as diabetes and obesity. Mg deficiency is often reported in DM [1,11,23,24,26,266] and obesity [105,267,268], both of which are high risk factors of developing CVD. Strong associations are reported between metabolic diseases and hypomagnesemia, inflammation, and oxidative stress [152]. Type 1 DM patients show Mg deficiency, and Mg hydroxide (Mg(OH)_2_, 500 mg/day for 21 weeks) improves insulin resistance [23] in part because of Mg upregulation of Akt2 and insulin receptor substrate 1 [183]. A meta-analysis study of randomized, double-blind controlled trials of 370 patients with type 2 DM shows that oral Mg supplementation at a median dose of 360 mg/day for a median duration of 12 weeks (4–16 weeks) is effective in reducing fasting plasma glucose levels and increasing high-density lipoprotein (HDL) cholesterol levels [266]. Long-term Mg supplementation significantly improves homeostatic model assessment of insulin resistance (HOMA-IR) index and fasting glucose in both diabetic and non-diabetic patients [269]. For example, oral MgCl_2_ (26 mmol/day for three months) shows significant improvement of insulin resistance with reduced HOMA-IR index, compared with placebo [97]. Oral MgCl_2_ supplementation of pre-diabetic patients with hypomagnesemia improves glycemic control with lower fasting and post-load glucose, and higher HDL cholesterol and serum Mg after four months of treatment [270]. A possible mechanism of Mg regulation of insulin signaling has been proposed in Figure 4.

Mg supplementation has shown important therapeutic effects in hypertension [15,16,271] and stroke [17,18]. Mg participates in the metabolism of l-arginine-NO system, essential fatty acids and eicosanoids. With Mg deficiency, the beneficial products such NO and resolvins are suppressed, while pro-inflammatory cytokines are elevated [103]. This can result in inflammation and vasoconstriction, cause high blood pressure and platelet aggregation [272], and induce hypertriglyceridemia and pro-atherogenic changes in lipoprotein metabolism [273,274], leading to the development of atherosclerosis and stroke. Mg supplementation has shown efficacy to treat vascular disease. For instance, oral Mg pidolate (600 mg/daily for 12 weeks) significantly reduced ambulatory blood pressure in patients with mild hypertension, accompanied by increased serum and intracellular Mg levels [271]. Dickinson et al. reviewed 12 randomized controlled trials and reported that diastolic but not systolic blood pressure is significantly decreased by Mg supplementation [15]. A recent meta-analysis study of 22 trials shows that Mg supplementation (120–973 mg/day) slightly but significantly decreases both systolic and diastolic blood pressure in a dose-dependent manner [7]. Mg can inhibit tissue transglutaminase and lysyl oxidase, both of which are associated with hypertension and atherosclerosis [275]. Mg can be used to reduce thrombotic complications. The Mg transporter TRPM7 has been found to be a key modulator of phospholipase C and the platelet Ca transient. High serum Mg inhibits TRPM7 activity. Blockade of TRPM7 kinase activity causes a significant defect in platelet aggregation and exhibits protective effects from ischemic stroke [276]. Mg inhibition of mitochondrial ROS production plays a protective role in carotid artery stenosis [141].

Mg deficiency has been reported to contribute to other vascular disorders such as pre-eclampsia and eclampsia [277,278,279,280,281]. IV MgSO_4_ became a standard treatment for pre-eclampsia and eclampsia seven decades ago [81,277,278,279] and the pharmacokinetic properties have been studied [282,283]. MgSO_4_ decreases the risk of eclampsia to half and reduces the risk of maternal death [280]. Mg supplementation helps decrease oxidative stress, suppress inflammation, reduce clotting factors [284], and increase the expression of calcitonin gene-related peptides and substance P that have vasodilatory effects and improve pre-eclampsia in women [85,285,286].

### 3.4. Gender Differences in Mg Therapy

Clinical studies have shown gender differences in Mg deficiency and associated diseases. For example, lower serum Mg is associated with DM and obesity in women but not in men [267,268]. Lower serum Mg levels are associated with a higher risk of cardiovascular mortality and all-cause mortality in female patients but not in the male subgroup [287,288,289], and the protective effect of Mg intake against total cardiovascular disease mortality risk is also observed most strongly in women [290]. IV MgCl_2_ shortens the atrial effective refractory period in women but prolongs the interval in men [291]. Mg intake is associated with the reduction in the systematic inflammation markers plasma CRP and E-selectin in women [137]. Generally, females seem more vulnerable to Mg deficiency. The mechanism of gender differences is unclear.

## 4. Mg Treatment: Routes, Chemical Formulations, Doses, and Duration

Mg supplementation is normally administrated via mouth, IV or intramuscular injection. Table 2 lists Mg supplementations that are effective in some clinical studies on treatment of HF, TdP, AF, VA, acute MI and hypertension in different salt formulations, routes of delivery, doses and treatment duration. The serum Mg level was often elevated after Mg treatment, no matter whether the patients had normal Mg levels or hypomagnesemia. Oral administration normally lasts longer (weeks to months, even a year in [197]) and is suitable for patients with low serum Mg levels and chronic diseases that are associated with Mg deficiency. Daily Mg supplementation can improve Mg deficiency-induced oxidative stress and inflammation with little side effects. Common oral Mg formulations include Mg l-lactate dehydrate, Mg gluconate, MgCl_2_, and Mg l-aspartate hydrochloride [292]. Organic formulations of Mg supplementation such as gluconate, lactate, and aspartate have been reported to have better [293] or equivalent [294] bioavailability compared with inorganic salts such as MgCl_2_ and MgSO_4_. Mg oxide and carbonate show extremely low bioavailability, probably because of their low solubility [292]. The oral dose applied in clinic studies listed in Table 2 ranges from 1.0 to 16 mmol Mg/day with a duration from three weeks to one year. Oral Mg therapy has some limitations, however, because of slow absorption and a simultaneous increase in urinary clearance of Mg. For example, oral Mg L-lactate does not elevate serum or intracellular Mg in >80% of patients who have intracellular Mg deficiency [295]. 

The IV route of Mg supplementation mainly uses the inorganic salts MgSO_4_ and MgCl_2_ to acutely treat patients with arrhythmias. The dose ranges from 0.1–100 mmol/h. A high dose but short treatment for only a few minutes is often given to patients as the first bolus to boost Mg levels, followed by a significantly lower dose but lasting for hours as a prophylactic treatment. For example, in the LIMIT-2 trial, IV MgSO_4_ infusion was administrated at 96 mmol/h for 5 min followed by 2.7 mmol/h for 24 h to treat acute MI patients [192]. The patient average serum Mg level increased from 0.80 ± 0.10 to 1.55 ± 0.10 mmol/L after the first dose and was maintained at 1.80 ± 0.15 mmol/L after the second dose. The treatment significantly decreased left ventricular failure and morality [192].

Mg supplementation has beneficial effects when combined with other treatments. For example, oral Mg supplementation has been used concomitantly with antiarrhythmic drugs [201]. IV Mg administration improves the efficacy of ibutilide in converting AF or flutter [306,307]. IV Mg is considered as a safe and effective adjunct to digoxin in controlling the ventricular response in AF in a meta-analysis of 515 patients with AF [203]. Early Mg therapy, when conjoined with reperfusion therapy, is reported to decrease the mortality after MI and the occurrence of HF [192,308]. Mg orotate used as adjuvant therapy in severe NYHA IV HF patients shows increased survival rate and improves clinical symptoms when compared with the placebo [197]. For HF patients using diuretics, hypomagnesemia also predisposes to hypokalemia. It is therefore critical to maintain both Mg and K levels by using either Mg/K supplementation or K- and Mg-sparing diuretics that inhibit renal K and Mg excretion (such as spironolactone, triamterene and amiloride) [309,310,311]. 

Adverse side effects such as gastrointestinal distress, diarrhea, and nausea have been reported with Mg supplementation for both oral and IV administration [203,266]. For IV infusion, this could be eliminated by slower infusion. For example, slow infusion of IV MgSO_4_ (2.5 mM/h for 2 h and 1.5 mM/h for 1.5 h) was used to treat patients with TdP, and patients showed no side effects [41]. For oral administration, smaller doses with multiple times per day may lead to less side effects.

Another way to maintain a normal Mg level is to increase dietary Mg intake. Recommended dietary allowances of Mg for adults are 400–420 mg for men and 310–320 mg for women [312]. In a study of young American adults (4637 Americans of 18–30 years old), Mg intake is reported to be inversely associated with the incidence of metabolic syndrome regardless of gender and race [21]. People who had the highest daily Mg intake (~191 mg Mg/1000 kcal) had significantly higher HDL cholesterol, lower blood glucose, lower fasting insulin levels, and lower blood pressure, compared with people who had the lowest daily Mg intake (~96 mg/1000 kcal) [21]. In a meta-analysis of 40 prospective cohort studies with more than one million patients, Fang et al. reported that increasing dietary Mg intake (per 100 mg/day increment) is linked to a 22% reduction in the risk of HF, as well as reduced risks of all-cause mortality, stroke, and type 2 DM [313]. Mg-rich food includes dark leafy greens (spinach, Swiss chard, kale, collard greens, turnip greens), vegetables (acorn squash, artichokes, okra, sweet corn, potato), nuts (almond, cashew, peanuts, and Brazil nuts), seeds (flax, pumpkin, sesame seeds, chia seeds), legumes (black beans, kidney beans, soybeans, lima beans, lentils, chickpeas, and green peas), fatty fish (salmon, tuna, mackerel, and halibut), whole grains, fruits (bananas, dried figs, guavas, avocados, kiwi, papaya, berries, cantaloupe, and grapefruit), dark chocolate, and yogurt. Most of the popular diets such as DASH (dietary approaches to stop hypertension) diet, Mediterranean diet, MIND diet (combination of the Mediterranean and DASH diet), Mayo Clinic diet, and vegetarian diet emphasize eating more vegetables, whole grains, and fruits that are all Mg-rich food [314]. Some diets such as the Mediterranean diet also encourage nuts, seeds, legumes, and fatty fish that contain higher amounts of Mg. For example, daily Mg intake in the DASH diet is ~500 mg [315], which meets the recommended dietary allowance. These diets also discourage processed food with high saturated fat and sugar but low Mg. 

## 5. Limitations and Controversial Reports of Mg Supplementation

Not all reports of Mg use are positive and not all preparations of Mg have the same effect. Despite efficacy on some arrhythmias, Mg showed no statistically significant effect on monomorphic VT, shock-resistant VF, and postoperative AF incidence [316,317,318]. Mg showed little effect on implantable cardioverter defibrillator (ICD) therapy for ischemic cardiomyopathy. For instance, oral Mg L-lactate treatment of patients with ICDs who had Mg deficiency did not reduce the occurrence of arrhythmias in ICD patients and had little impact on the health-related quality of life [319]. While several studies show that IV Mg supplementation (MgSO_4_ and MgCl_2_) improved mortality in acute MI patients [192,218,219,221], Feldstedt et al. observed no improvement with IV MgCl_2_ on either the in-hospital or the follow-up mortality after acute myocardial infarction [320]. Moreover, Mg infusion was accompanied by a significantly increased incidence of atrioventricular conduction disturbances. Eichhorn et al. reported no correlation between serum Mg levels and survival of HF patients [321]. These controversies could result from variances in Mg treatments (different salts, routes, doses, treatment timing and durations) or severity of disease. Nevertheless, it appears that Mg has limited efficacy to treat acute reentrant rhythms such as ventricular tachycardia or atrial fibrillation [318,322,323] and that Mg may not be as efficacious in ischemic heart disease [319,320], where the arrhythmic substrate may be based more on structural issues and reentrant mechanisms. 

## 6. Conclusions

Mg deficiency is common in CVD, and Mg supplementation has shown antioxidant and anti-inflammatory properties in patients with and without Mg deficiency. Mg supplementation is well tolerated with few side effects. Therefore, it may represent a reasonable additional therapy for many CVDs.

## Figures and Tables

**Table 1:** Mg deficiency activates inflammation with increased inflammatory markers reported in clinical and animal studies.

Increased Inflammatory Markers	Tested Targets	Reference
CRP	Human HF	[140]
	Postmenopausal women	[146]
MCP-1	Human carotid artery stenosis	[141]
	Human dermal microvascular cells	[144]
	Mouse hearts	Our unpublished work
substance P	Rats	[147,148]
	Rats with cardiomyopathic lesions	[149]
	Mouse megakaryocytes and lymphocytes	[66]
IL-1	Human umbilical vein endothelial cells	[136]
	Mouse osteoclasts	[66]
	Hamsters and rats with cardiac lesions	[134]
	Rat bone cells	[148]
IL-6	Postmenopausal women	[146]
	Aging rats with CVD	[142,143]
	Hamsters and rats with cardiac lesions	[134]
IL-8	Human dermal microvascular cells	[144]
	Hamsters and rats with cardiac lesions	[134]
TNF-α	Type 2 diabetes patients	[150]
	Postmenopausal women	[146]
	Aging rats with CVD	[142,143]
	Hamsters and rats with cardiac lesions	[134]
	Rat bone cells	[148]
	Mouse osteoclasts	[66]
elevated cytokines, leukocyte and macrophage activation	Rats	[93,135]

**Table 2:** Mg treatments in different salt forms, routes, doses, and treatment durations that present protective effects in clinical studies on heart diseases. Serum Mg levels before and after Mg treatment are listed if tested in the studies. Serum Mg values are mean ± SD unless otherwise stated.

Salts	Route	Dose	Treatment Duration	Treating Diseases	Serum Mg (mmol/L)		Reference
					Before Treatment	After Treatment	
MgSO_4_	Oral IV	5.9 mmol/day MgCO_3_ 22 mmol/h	8 days 12 h on day 9	NYHA II-IV HF-VA	0.66 ± 0.14	0.74 ± 0.13	[10]
	IV	0.1 mmol/h	1 h	NYHA II-IV HF-VA	0.74 ± 0.0	1.15 ± 0.16	[196]
	IV	1.67 mmol/h	24 h	NYHA II-III HF-QT variability, ischemic HF	0.85 ± 0.02 ^1^	1.09 ± 0.07 ^1^	[194]
	IV	1st bolus 30 mmol/h 2nd bolus 5 mmol/h	20 min 2 h	AF	0.85 ± 0.10	NA	[207]
	IV	1st bolus 66 mmol/h 2nd bolus 11 mmol/h	15 min 6 h	New-onset AF	1.1 ± 0.4 ^1^	NA	[296]
	IV	0.15 mmol/kg BW 0.10 mmol/kg BW	10 min 10 h	Chronic AF	0.90 ± 0.09	1.62 ± 0.29	[297]
	IV	12–40 mmol total	20 min–24 h	Acute onset AF	normal	NA	[203]
	IV	24 mmol/h	20 min, twice in 6 h for 3 days	Postoperative AT	0.85 ± 0.08	0.95 ± 0.09	[198]
	IV	2.7 mmol/h	24 h	AMI-VA and mortality	0.80 ± 0.01 ^1^	1.51 ± 0.03 ^1^	[219]
	IV	2.8 mmol/h	24 h	AMI-VT	0.8 ± 0.2	1.0 ± 0.1	[298]
	IV	3.8 mmol/h	48 h	AMI-HF and mortality	NA	NA	[221]
	IV	60 mmol/h	20 min	AMI-VA	0.92 ± 0.02 ^1^	1.07 ± 0.02 ^1^	[220]
	IV	1st 48 mmol/h, 2nd 2.6 mmol/h	10 min 24 h	AMI-VA	0.78 (0.61–0.93) ^2^	1.30 (1.11–1.74) ^2^	[224]
	IV	1st 96 mmol/h 2nd 2.7 mmol/h	5 min 24 h	AMI-HF and mortality	0.80 ± 0.10	1.55 ± 0.10 1.80 ± 0.15	[192]
	IV	1.5–10 mmol/h	1.7–11.1 h	TdP	0.94 ± 0.08	1.56 ± 0.43	[200]
	IV	25 mmol/h 15 mmol/h	2 h 1.5 h, twice a day for 3–4 days	TdP	0.98 ± 0.11	1.08 ± 0.08	[41]
	IV	1st day 20 mmol/h then 5 mmol/h	~50 min 50 min, once a day for 3–4 days	TdP	normal	NA	[299]
	IV	33 mmol/h 1.5 mmol/h	30 min 24 h	VT	NA	NA	[300]
	IV	0.21–0.42 mmol/kg BW	intraoperation	Postoperative arrhythmias	NA	NA	[199]
MgCl_2_	Oral	15.8 mmol/day	6 w	NYHA II-IV HF-VA	0.87 ± 0.05	0.92 ± 0.05	[195]
	IV	1st bolus 5 mmol/h 2nd bolus 1.1 mmol/h 3rd bolus 0.5 mmol/h	6 h 18 h 24 h	AMI-VA and mortality	0.75 (0.56–0.98) ^2^	1.23 (0.77–2.27) ^2^	[218]
	IV	72 mmol/h	10 min	SVA	0.78 ± 0.03 ^1^	1.52 ± 0.08 ^1^	[301]
	IV	42 mmol/h	30 min	Postoperative VA	0.82 ± 0.02	NA	[302]
	IV	1 mmol/h	48 h	Postoperative SVA	0.82 ± 0.05	1.20 ± 0.25	[303]
Mg oxide	Oral	9.9 mmol/day	12 w	AF	0.87 ± 0.06	0.90 ± 0.06	[47]
Mg citrate	Oral	1.4 mmol/day	5 w	HF – heart rate variation	0.78 ± 0.04	0.89 ± 0.06	[57]
Mg gluconate	Oral	7.2 mmol/day	~10 w	Pregnancy-induced hypertension	NA	NA	[304]
Mg glutamate	IV	2.6 mmol	twice	TdP	0.79 ± 0.10	1.87 ± 0.50	[305]
Mg-DL-hydrogen-aspartate	Oral	6 mmol/day	3 w	VA	0.85 ± 0.03	0.88 ± 0.04	[46]
Mg L-lactate	Oral	21 mmol/day	12–24 w	Hypertension of ICD patients	0.91 ± 0.08	0.99 ± 0.08	[295]
Mg orotate	Oral	1.00 ± 0.04 mmol/day	1 year	NYHY IV HF-survival and symptoms	NA	NA	[197]

^1^ Value = mean±SEM; ^2^ median (range). Abbreviations: min, minute/minutes; h, hour/hours; w, week/weeks; AF, atrial fibrillation; AT, atrial tachycardia; AMI, acute myocardial infarction; BW, body weight; HF, heart failure; NA, not available; SVA, supraventricular arrhythmia; TdP, torsades de pointes; VA, ventricular arrhythmias; VT, ventricular tachycardia; IV, intravenous.

## References

[1] Liu M., Jeong E.-M., Liu H., Xie A., So E.Y., Shi G., Jeong G.E., Zhou A., Dudley S.C. (2019). Magnesium supplementation improves diabetic mitochondrial and cardiac diastolic function. JCI Insight.

[2] Benjamin E.J., Muntner P., Alonso A., Bittencourt M.S., Callaway C.W., Carson A.P., Chamberlain A.M., Chang A.R., Cheng S., Das S.R. (2019). Heart disease and stroke statistics-2019 update: A report from the American Heart Association. Circulation.

[3] Centers for Disease Control and Prevention (2020). Underlying cause of death. National Center for Health Statistics: 1999–2018.

[4] Douban S., Brodsky M.A., Whang D.D., Whang R. (1996). Significance of magnesium in congestive heart failure. Am. Heart J..

[5] Milionis H.J., Alexandrides G.E., Liberopoulos E.N., Bairaktari E.T., Goudevenos J., Elisaf M.S. (2002). Hypomagnesemia and concurrent acid–base and electrolyte abnormalities in patients with congestive heart failure. Eur. J. Heart Fail..

[6] Tangvoraphonkchai K., Davenport A. (2018). Magnesium and cardiovascular disease. Adv. Chronic Kidney Dis..

[7] DiNicolantonio J.J., Liu J., O’Keefe J.H. (2018). Magnesium for the prevention and treatment of cardiovascular disease. Open Heart.

[8] Al Alawi A.M., Majoni S.W., Falhammar H. (2018). Magnesium and human health: Perspectives and research directions. Int. J. Endocrinol..

[9] Schwinger R.H., Erdmann E. (1992). Heart failure and electrolyte disturbances. Methods Find. Exp. Clin. Pharmacol..

[10] Ceremużyński L., Gębalska J., Wołk R., Makowska E. (2000). Hypomagnesemia in heart failure with ventricular arrhythmias. Beneficial effects of magnesium supplementation. J. Intern. Med..

[11] Gommers L.M., Hoenderop J.G., Bindels R.J., de Baaij J.H. (2016). Hypomagnesemia in type 2 diabetes: A vicious circle?. Diabetes.

[12] Shechter M. (2010). Magnesium and cardiovascular system. Magnes. Res..

[13] Reffelmann T., Ittermann T., Dörr M., Völzke H., Reinthaler M., Petersmann A., Felix S.B. (2011). Low serum magnesium concentrations predict cardiovascular and all-cause mortality. Atherosclerosis.

[14] Severino P., Netti L., Mariani M.V., Maraone A., D’Amato A., Scarpati R., Infusino F., Pucci M., Lavalle C., Maestrini V. (2019). Prevention of cardiovascular disease: Screening for magnesium deficiency. Cardiol. Res. Pract..

[15] Dickinson H.O., Nicolson D.J., Campbell F., Cook J.V., Beyer F.R., Ford G.A., Mason J. (2006). Magnesium supplementation for the management of essential hypertension in adults. Cochrane Database Syst. Rev..

[16] Kass L., Weekes J., Carpenter L. (2012). Effect of magnesium supplementation on blood pressure: A meta-analysis. Eur. J. Clin. Nutr..

[17] Altura B.M., Kostellow A.B., Zhang A., Li W., Morrill G.A., Gupta R.K., Altura B.T. (2003). Expression of the nuclear factor-kB and proto-oncogenes c-fos and c-jun are induced by low extracellular Mg^2+^ in aortic and cerebral vascular smooth muscle cells: Possible links to hypertension, atherogenesis, and stroke. Am. J. Hypertens..

[18] Zhao B., Hu L., Dong Y., Xu J., Wei Y., Yu D., Xu J., Zhang W. (2019). The effect of magnesium intake on stroke incidence: A systematic review and meta-analysis with trial sequential analysis. Front. Neurol..

[19] Volpe S.L. (2013). Magnesium in disease prevention and overall health. Adv. Nutr..

[20] De Baaij J.H., Hoenderop J.G., Bindels R.J. (2015). Magnesium in man: Implications for health and disease. Physiol. Rev..

[21] He K., Liu K., Daviglus M.L., Morris S.J., Loria C.M., Van Horn L., Jacobs D.R., Savage P.J. (2006). Magnesium intake and incidence of metabolic syndrome among young adults. Circulation.

[22] Belin R.J., He K. (2007). Magnesium physiology and pathogenic mechanisms that contribute to the development of the metabolic syndrome. Magnes. Res..

[23] Sjögren A., Florén C.H., Nilsson A. (1988). Oral administration of magnesium hydroxide to subjects with insulin-dependent diabetes mellitus: Effects on magnesium and potassium levels and on insulin requirements. Magnesium.

[24] Djurhuus M.S., Klitgaard N.A., Pedersen K.K., Blaabjerg O., Altura B.M., Altura B.T., Henriksen J.E. (2001). Magnesium reduces insulin-stimulated glucose uptake and serum lipid concentrations in type 1 diabetes. Metabolism.

[25] Song Y., Manson J.E., Buring J.E., Liu S. (2004). Dietary magnesium intake in relation to plasma insulin levels and risk of type 2 diabetes in women. Diabetes Care.

[26] Martins I.J. (2017). Magnesium deficiency and induction of NAFLD and type 3 diabetes in Australasia. Australas. Med. J..

[27] Ascherio A., Rimm E.B., Giovannucci E.L., Colditz G.A., Rosner B., Willett W.C., Sacks F., Stampfer M.J. (1992). A prospective study of nutritional factors and hypertension among US men. Circulation.

[28] Ascherio A., Hennekens C., Willett W.C., Sacks F., Rosner B., Manson J., Witteman J., Stampfer M.J. (1996). Prospective study of nutritional factors, blood pressure, and hypertension among US women. Hypertension.

[29] Haigney M.C., Wei S., Kääb S., Griffiths E., Berger R., Tunin R., Kass D., Fisher W.G., Silver B., Silverman H. (1998). Loss of cardiac magnesium in experimental heart failure prolongs and destabilizes repolarization in dogs. J. Am. Coll. Cardiol..

[30] Schimatschek H.F., Rempis R. (2001). Prevalence of hypomagnesemia in an unselected German population of 16,000 individuals. Magnes. Res..

[31] Guo W., Nazim H., Liang Z., Yang D. (2016). Magnesium deficiency in plants: An urgent problem. Crop J..

[32] Olza J., Aranceta-Bartrina J., Gonzalez-Gross M., Ortega R.M., Serra-Majem L., Varela-Moreiras G., Gil A. (2017). Reported dietary intake, disparity between the reported consumption and the level needed for adequacy and food sources of calcium, phosphorus, magnesium and vitamin D in the Spanish population: Findings from the ANIBES Study. Nutrients.

[33] Moshfegh A., Goldman J., Ahuja J., Rhodes D., Lacomb R. What We Eat in America, NHANES 2005–2006, Usual Nutrient Intakes from Food and Water Compared to 1997 Dietary Reference Intakes for Vitamin D, Calcium, Phosphorus, and Magnesium. https://www.ars.usda.gov/research/publications/publication/?seqNo115=243279.

[34] Costello R.B., Elin R.J., Rosanoff A., Wallace T.C., Guerrero-Romero F., Hruby A., Lutsey P.L., Nielsen F.H., Rodriguez-Moran M., Song Y. (2016). Perspective: The case for an evidence-based reference interval for serum magnesium: The time has come. Adv. Nutr..

[35] Nielsen F.H. (2018). Magnesium deficiency and increased inflammation: Current perspectives. J. Inflamm. Res..

[36] Ford E.S., Mokdad A.H. (2003). Dietary magnesium intake in a national sample of US adults. J. Nutr..

[37] Corica F., Corsonello A., Ientile R., Cucinotta D., Di Benedetto A., Perticone F., Dominguez L.J., Barbagallo M. (2006). Serum ionized magnesium levels in relation to metabolic syndrome in type 2 diabetic patients. J. Am. Coll. Nutr..

[38] Taveira T.H., Ouellette D., Gulum A., Choudhary G., Eaton C.B., Liu S., Wu W.C. (2016). Relation of magnesium intake with cardiac function and heart failure hospitalizations in black adults: The Jackson Heart Study. Circ. Heart Fail..

[39] Gottlieb S.S. (1989). Importance of magnesium in congestive heart failure. Am. J. Cardiol..

[40] Chrysant S.G., Chrysant G.S. (2019). Association of hypomagnesemia with cardiovascular diseases and hypertension. Int. J. Cardiol. Hypertens..

[41] Perticone F., Adinolfi L., Bonaduce D. (1986). Efficacy of magnesium sulfate in the treatment of torsade de pointes. Am. Heart J..

[42] Gupta A., Lawrence A.T., Krishnan K., Kavinsky C.J., Trohman R.G. (2007). Current concepts in the mechanisms and management of drug-induced QT prolongation and torsade de pointes. Am. Heart J..

[43] Fernando H.C., Jaklitsch M.T., Walsh G.L., Tisdale J.E., Bridges C.D., Mitchell J.D., Shrager J.B. (2011). The Society of Thoracic surgeons practice guideline on the prophylaxis and management of atrial fibrillation associated with general thoracic surgery: Executive summary. Ann. Thorac. Surg..

[44] Frendl G., Sodickson A.C., Chung M.K., Waldo A.L., Gersh B.J., Tisdale J.E., Calkins H., Aranki S., Kaneko T., Cassivi S. (2014). 2014 AATS guidelines for the prevention and management of perioperative atrial fibrillation and flutter for thoracic surgical procedures. J. Thorac. Cardiovasc. Surg..

[45] Markovits N., Kurnik D., Halkin H., Margalit R., Bialik M., Lomnicky Y., Loebstein R. (2016). Database evaluation of the association between serum magnesium levels and the risk of atrial fibrillation in the community. Int. J. Cardiol..

[46] Zehender M., Meinertz T., Faber T., Caspary A., Jeron A., Bremm K., Just H. (1997). Antiarrhythmic effects of increasing the daily intake of magnesium and potassium in patients with frequent ventricular arrhythmias. Magnesium in Cardiac Arrhythmias (MAGICA) Investigators. J. Am. Coll. Cardiol..

[47] Lutsey P., Chen L., Eaton A., Jaeb M., Rudser K., Neaton J., Alonso A. (2018). A pilot randomized trial of oral magnesium supplementation on supraventricular arrhythmias. Nutrients.

[48] Horner S.M. (1992). Efficacy of intravenous magnesium in acute myocardial infarction in reducing arrhythmias and mortality. Meta-analysis of magnesium in acute myocardial infarction. Circulation.

[49] Ferrari R., Albertini A., Curello S., Ceconi C., Di Lisa F., Raddino R., Visioli O. (1986). Myocardial recovery during post-ischaemic reperfusion: Effects of nifedipine, calcium and magnesium. J. Mol. Cell. Cardiol..

[50] Gout E., Rébeillé F., Douce R., Bligny R. (2014). Interplay of Mg^2+^, ADP, and ATP in the cytosol and mitochondria: Unravelling the role of Mg^2+^ in cell respiration. Proc. Natl. Acad. Sci. USA.

[51] Yamanaka R., Tabata S., Shindo Y., Hotta K., Suzuki K., Soga T., Oka K. (2016). Mitochondrial Mg^2+^ homeostasis decides cellular energy metabolism and vulnerability to stress. Sci. Rep..

[52] Sreedhara A., Cowan J.A. (2002). Structural and catalytic roles for divalent magnesium in nucleic acid biochemistry. BioMetals.

[53] Sharikabad M.N., Østbye K.M., Brørs O. (2001). Increased [Mg^2+^] reduces Ca^2+^ influx and disruption of mitochondrial membrane potential during reoxygenation. Am. J. Physiol. Heart Circ. Physiol..

[54] Racay P. (2008). Effect of magnesium on calcium-induced depolarisation of mitochondrial transmembrane potential. Cell Biol. Int..

[55] Buda S., Stompor T., Sulowicz W., Kopec J., Szymczakiewicz-Multanowska A., Janion M. (2000). The impact of changes in levels of calcium, phosphate and magnesium during hemodialysis on autonomic system reactivity as measured by heart rate variability analysis. Przegl. Lek..

[56] Mubagwa K., Gwanyanya A., Zakharov S., Macianskiene R. (2007). Regulation of cation channels in cardiac and smooth muscle cells by intracellular magnesium. Arch. Biochem. Biophys..

[57] Almoznino-Sarafian D., Sarafian G., Berman S., Shteinshnaider M., Tzur I., Cohen N., Gorelik O. (2009). Magnesium administration may improve heart rate variability in patients with heart failure. Nutr. Metab. Cardiovasc. Dis..

[58] Zhao M., Feng R., Shao D., Liu S., Lei M., Wang H., Sun X., Guo F., Hu H., Kameyama M. (2015). Mg^2+^-dependent facilitation and inactivation of L-type Ca^2+^ channels in guinea pig ventricular myocytes. J. Pharmacol. Sci..

[59] Finley N., Dvoretsky A., Rosevear P.R. (2000). Magnesium-calcium exchange in cardiac troponin C bound to cardiac troponin I. J. Mol. Cell. Cardiol..

[60] Martin S.R., Masino L., Bayley P.M. (2000). Enhancement by Mg^2+^ of domain specificity in Ca^2+^-dependent interactions of calmodulin with target sequences. Protein Sci..

[61] Carvil P., Cronin J. (2010). Magnesium and implications on muscle function. Strenth Cond. J..

[62] Zhang X., Li Y., Del Gobbo L.C., Rosanoff A., Wang J., Zhang W., Song Y. (2016). Effects of magnesium supplementation on blood pressure: A meta-analysis of randomized double-blind placebo-controlled trials. Hypertension.

[63] Romani A.M., Matthews V.D., Scarpa A. (2000). Parallel stimulation of glucose and Mg^2+^ accumulation by insulin in rat hearts and cardiac ventricular myocytes. Circ. Res..

[64] Feng J., Wang H., Jing Z., Wang Y., Cheng Y., Wang W., Sun W. (2019). Role of magnesium in type 2 diabetes mellitus. Biol. Trace Elem. Res..

[65] Tucker K.L., Hannan M.T., Chen H., Cupples L.A., Wilson P.W., Kiel D.P. (1999). Potassium, magnesium, and fruit and vegetable intakes are associated with greater bone mineral density in elderly men and women. Am. J. Clin. Nutr..

[66] Rude R.K., Gruber H.E., Wei L.Y., Frausto A., Mills B.G. (2003). Magnesium deficiency: Effect on bone and mineral metabolism in the mouse. Calcif. Tissue Int..

[67] Erem S., Atfi A., Razzaque M.S. (2019). Anabolic effects of vitamin D and magnesium in aging bone. J. Steroid Biochem. Mol. Biol..

[68] Soar J., Perkins G.D., Abbas G., Alfonzo A., Barelli A., Bierens J.J., Brugger H., Deakin C.D., Dunning J., Georgiou M. (2010). European Resuscitation Council Guidelines for Resuscitation 2010 Section 8. Cardiac arrest in special circumstances: Electrolyte abnormalities, poisoning, drowning, accidental hypothermia, hyperthermia, asthma, anaphylaxis, cardiac surgery, trauma, pregnancy, electrocution. Resuscitation.

[69] Pham P.C., Pham P.A., Pham S.V., Pham P.T., Pham P.M., Pham P.T. (2014). Hypomagnesemia: A clinical perspective. Int. J. Nephrol. Renovasc. Dis..

[70] Watanabe M., Konishi M. (2001). Intracellular calibration of the fluorescent Mg^2+^ indicator furaptra in rat ventricular myocytes. Pflugers Arch..

[71] Tashiro M., Inoue H., Konishi M. (2013). Magnesium homeostasis in cardiac myocytes of Mg-deficient rats. PLoS ONE.

[72] Touyz R.M. (2008). Transient receptor potential melastatin 6 and 7 channels, magnesium transport, and vascular biology: Implications in hypertension. Am. J. Physiol. Heart Circ. Physiol..

[73] Tashiro M., Inoue H., Konishi M. (2014). Physiological pathway of magnesium influx in rat ventricular myocytes. Biophys. J..

[74] Arjona F.J., Chen Y.X., Flik G., Bindels R.J., Hoenderop J.G. (2013). Tissue-specific expression and in vivo regulation of zebrafish orthologues of mammalian genes related to symptomatic hypomagnesemia. Pflug. Arch..

[75] Kolisek M., Zsurka G., Samaj J., Weghuber J., Schweyen R.J., Schweigel M. (2003). Mrs2p is an essential component of the major electrophoretic Mg^2+^ influx system in mitochondria. EMBO J..

[76] Mastrototaro L., Smorodchenko A., Aschenbach J.R., Kolisek M., Sponder G. (2016). Solute carrier 41A3 encodes for a mitochondrial Mg^2+^ efflux system. Sci. Rep..

[77] Pilchova I., Klacanova K., Tatarkova Z., Kaplan P., Racay P. (2017). The involvement of Mg^2+^ in regulation of cellular and mitochondrial functions. Oxid. Med. Cell. Longev..

[78] Romani A.M.P. (2011). Cellular magnesium homeostasis. Arch. Biochem. Biophys..

[79] Goytain A., Quamme G.A. (2005). Functional characterization of human SLC41A1, a Mg^2+^ transporter with similarity to prokaryotic MgtE Mg^2+^ transporters. Physiol. Genom..

[80] Zhou H., Clapham D.E. (2009). Mammalian MagT1 and TUSC3 are required for cellular magnesium uptake and vertebrate embryonic development. Proc. Natl. Acad. Sci. USA.

[81] Shahi A., Aslani S., Ataollahi M., Mahmoudi M. (2019). The role of magnesium in different inflammatory diseases. Inflammopharmacology.

[82] Liu M., Liu H., Xie A., Kang G.J., Feng F., Zhou X., Zhao Y., Dudley S.C. (2020). Magnesium deficiency causes reversible diastolic and systolic cardiomyopathy. Biophys. J..

[83] He Y., Yao G., Savoia C., Touyz R.M. (2005). Transient receptor potential melastatin 7 ion channels regulate magnesium homeostasis in vascular smooth muscle cells: Role of angiotensin II. Circ. Res..

[84] Sontia B., Montezano Augusto C.I., Paravicini T., Tabet F., Touyz Rhian M. (2008). Downregulation of renal TRPM7 and increased inflammation and fibrosis in aldosterone-infused mice. Hypertension.

[85] Yogi A., Callera G.E., O’Connor S., Antunes T.T., Valinsky W., Miquel P., Montezano A.C.I., Perraud A.-L., Schmitz C., Shrier A. (2013). Aldosterone signaling through transient receptor potential melastatin 7 cation channel (TRPM7) and its α-kinase domain. Cell. Signal..

[86] Weglicki W., Quamme G., Tucker K., Haigney M., Resnick L. (2005). Potassium, magnesium, and electrolyte imbalance and complications in disease management. Clin. Exp. Hypertens..

[87] Liamis G., Rodenburg E.M., Hofman A., Zietse R., Stricker B.H., Hoorn E.J. (2013). Electrolyte disorders in community subjects: Prevalence and risk factors. Am. J. Med..

[88] Tham Y.K., Bernardo B.C., Ooi J.Y.Y., Weeks K.L., McMullen J.R. (2015). Pathophysiology of cardiac hypertrophy and heart failure: Signaling pathways and novel therapeutic targets. Arch. Toxicol..

[89] Panov A., Scarpa A. (1996). Mg^2+^ control of respiration in isolated rat liver mitochondria. Biochemistry.

[90] Rodríguez-Zavala J.S., Moreno-Sánchez R. (1998). Modulation of oxidative phosphorylation by Mg^2+^ in rat heart mitochondria. J. Biol. Chem..

[91] Salaminia S., Sayehmiri F., Angha P., Sayehmiri K., Motedayen M. (2018). Evaluating the effect of magnesium supplementation and cardiac arrhythmias after acute coronary syndrome: A systematic review and meta-analysis. BMC Cardiovasc. Disord..

[92] Rayssiguier Y., Libako P., Nowacki W., Rock E. (2010). Magnesium deficiency and metabolic syndrome: Stress and inflammation may reflect calcium activation. Magnes. Res..

[93] Rayssiguier Y., Gueux E., Nowacki W., Rock E., Mazur A. (2006). High fructose consumption combined with low dietary magnesium intake may increase the incidence of the metabolic syndrome by inducing inflammation. Magnes. Res..

[94] Bo S., Durazzo M., Guidi S., Carello M., Sacerdote C., Silli B., Rosato R., Cassader M., Gentile L., Pagano G. (2006). Dietary magnesium and fiber intakes and inflammatory and metabolic indicators in middle-aged subjects from a population-based cohort. Am. J. Clin. Nutr..

[95] Tejero-Taldo M.I., Chmielinska J.J., Gonzalez G., Mak I.T., Weglicki W.B. (2004). N-methyl-D-aspartate receptor blockade inhibits cardiac inflammation in the Mg^2+^-deficient rat. J. Pharmacol. Exp. Ther..

[96] Hans C.P., Chaudhary D.P., Bansal D.D. (2002). Magnesium deficiency increases oxidative stress in rats. Indian J. Exp. Biol..

[97] Guerrero-Romero F., Tamez-Perez H.E., González-González G., Salinas-Martínez A.M., Montes-Villarreal J., Treviño-Ortiz J.H., Rodríguez-Morán M. (2004). Oral magnesium supplementation improves insulin sensitivity in non-diabetic subjects with insulin resistance. A double-blind placebo-controlled randomized trial. Diabetes Metab..

[98] Liu M., Gu L., Sulkin M.S., Liu H., Jeong E.M., Greener I., Xie A., Efimov I.R., Dudley S.C. (2013). Mitochondrial dysfunction causing cardiac sodium channel downregulation in cardiomyopathy. J. Mol. Cell. Cardiol..

[99] Dey S., DeMazumder D., Sidor A., Foster D.B., O’Rourke B. (2018). Mitochondrial ROS drive sudden cardiac death and chronic proteome remodeling in heart failure. Circ. Res..

[100] García N., Zazueta C., Aguilera-Aguirre L. (2017). Oxidative stress and inflammation in cardiovascular disease. Oxid. Med. Cell. Longev..

[101] Pignatelli P., Menichelli D., Pastori D., Violi F. (2018). Oxidative stress and cardiovascular disease: New insights. Kardiol. Pol..

[102] Hashimoto T., Nishi K., Nagasao J., Tsuji S., Oyanagi K. (2008). Magnesium exerts both preventive and ameliorating effects in an in vitro rat Parkinson disease model involving 1-methyl-4-phenylpyridinium (MPP+) toxicity in dopaminergic neurons. Brain Res..

[103] Das U.N. (2015). Nutritional factors in the prevention and management of coronary artery disease and heart failure. Nutrition.

[104] Zheltova A.A., Kharitonova M.V., Iezhitsa I.N., Spasov A.A. (2016). Magnesium deficiency and oxidative stress: An update. Biomedicine (Taipei).

[105] Morais J.B.S., Severo J.S., Santos L.R.d., de Sousa Melo S.R., de Oliveira Santos R., de Oliveira A.R.S., Cruz K.J.C., do Nascimento Marreiro D. (2017). Role of magnesium in oxidative stress in individuals with obesity. Biol. Trace Elem. Res..

[106] Celik N., Andiran N., Yilmaz A.E. (2011). The relationship between serum magnesium levels with childhood obesity and insulin resistance: A review of the literature. J. Pediatr. Endocrinol. Metab..

[107] Codoner-Franch P., Boix-Garcia L., Simo-Jorda R., Del Castillo-Villaescusa C., Maset-Maldonado J., Valls-Belles V. (2010). Is obesity associated with oxidative stress in children?. Int. J. Pediatr. Obes..

[108] Kubota T., Shindo Y., Tokuno K., Komatsu H., Ogawa H., Kudo S., Kitamura Y., Suzuki K., Oka K. (2005). Mitochondria are intracellular magnesium stores: Investigation by simultaneous fluorescent imagings in PC12 cells. Biochim. Biophys. Acta.

[109] Jeong E.M., Chung J., Liu H., Go Y., Gladstein S., Farzaneh-Far A., Lewandowski E.D., Dudley S.C. (2016). Role of mitochondrial oxidative stress in glucose tolerance, insulin resistance, and cardiac diastolic dysfunction. J. Am. Heart Assoc..

[110] Kramer J.H., Mišík V., Weglicki W.B. (1994). Magnesium-deficiency potentiates free radical production associated with postischemic injury to rat hearts: Vitamin E affords protection. Free Radical. Biol. Med..

[111] Shah N.C., Liu J.-P., Iqbal J., Hussain M., Jiang X.-C., Li Z., Li Y., Zheng T., Li W., Sica A.C. (2011). Mg deficiency results in modulation of serum lipids, glutathione, and NO synthase isozyme activation in cardiovascular tissues: Relevance to de novo synthesis of ceramide, serum Mg and atherogenesis. Int. J. Clin. Exp. Med..

[112] Kumar B.P., Shivakumar K. (1997). Depressed antioxidant defense in rat heart in experimental magnesium deficiency. Implications for the pathogenesis of myocardial lesions. Biol. Trace Elem. Res..

[113] Canet-Avilés R.M., Wilson M.A., Miller D.W., Ahmad R., McLendon C., Bandyopadhyay S., Baptista M.J., Ringe D., Petsko G.A., Cookson M.R. (2004). The Parkinson’s disease protein DJ-1 is neuroprotective due to cysteine-sulfinic acid-driven mitochondrial localization. Proc. Natl. Acad. Sci. USA.

[114] Björkblom B., Maple-Grødem J., Puno M.R., Odell M., Larsen J.P., Møller S.G. (2014). Reactive oxygen species-mediated DJ-1 monomerization modulates intracellular trafficking involving karyopherin β2. Mol. Cell. Biol..

[115] Blomeyer C.A., Bazil J.N., Stowe D.F., Dash R.K., Camara A.K.S. (2016). Mg^2+^ differentially regulates two modes of mitochondrial Ca^2+^ uptake in isolated cardiac mitochondria: Implications for mitochondrial Ca^2+^ sequestration. J. Bioenerg. Biomembr..

[116] Sponder G., Abdulhanan N., Frohlich N., Mastrototaro L., Aschenbach J.R., Rontgen M., Pilchova I., Cibulka M., Racay P., Kolisek M. (2018). Overexpression of Na^+^/Mg^2+^ exchanger SLC41A1 attenuates pro-survival signaling. Oncotarget.

[117] Salvi M., Bozac A., Toninello A. (2004). Gliotoxin induces Mg^2+^ efflux from intact brain mitochondria. Neurochem. Int..

[118] Chen Y., Wei X., Yan P., Han Y., Sun S., Wu K., Fan D. (2009). Human mitochondrial Mrs2 protein promotes multidrug resistance in gastric cancer cells by regulating p27, cyclin D1 expression and cytochrome C release. Cancer Biol. Ther..

[119] Bednarczyk P., Dolowy K., Szewczyk A. (2005). Matrix Mg^2+^ regulates mitochondrial ATP-dependent potassium channel from heart. FEBS Lett..

[120] Beavis A.D., Powers M.F. (1989). On the regulation of the mitochondrial inner membrane anion channel by magnesium and protons. J. Biol. Chem..

[121] Zoratti M., Szabò I. (1995). The mitochondrial permeability transition. Biochim. Biophys. Acta.

[122] Seo Y.W., Shin J.N., Ko K.H., Cha J.H., Park J.Y., Lee B.R., Yun C.W., Kim Y.M., Seol D.W., Kim D.W. (2003). The molecular mechanism of Noxa-induced mitochondrial dysfunction in p53-mediated cell death. J. Biol. Chem..

[123] Gorgoglione V., Laraspata D., Piana G.L., Marzulli D., Lofrumento N.E. (2007). Protective effect of magnesium and potassium ions on the permeability of the external mitochondrial membrane. Arch. Biochem. Biophys..

[124] La Piana G., Gorgoglione V., Laraspata D., Marzulli D., Lofrumento N.E. (2008). Effect of magnesium ions on the activity of the cytosolic NADH/cytochrome C electron transport system. FEBS J..

[125] Huang C.-Y., Hsieh Y.-L., Ju D.-T., Lin C.-C., Kuo C.-H., Liou Y.-F., Ho T.-J., Tsai C.-H., Tsai F.-J., Lin J.-Y. (2015). Attenuation of magnesium sulfate on CoCl_2_-induced cell death by activating ERK1/2/MAPK and inhibiting HIF-1a via mitochondrial apoptotic signaling suppression in a neuronal cell line. Chin. J. Physiol..

[126] Boelens A.D., Pradhan R.K., Blomeyer C.A., Camara A.K.S., Dash R.K., Stowe D.F. (2013). Extra-matrix Mg^2+^ limits Ca^2+^ uptake and modulates Ca^2+^ uptake–independent respiration and redox state in cardiac isolated mitochondria. J. Bioenerg. Biomembr..

[127] Li Y., Wang J., Yue J., Wang Y., Yang C., Cui Q. (2018). High magnesium prevents matrix vesicle-mediated mineralization in human bone marrow-derived mesenchymal stem cells via mitochondrial pathway and autophagy. Cell Biol. Int..

[128] Piskacek M., Zotova L., Zsurka G., Schweyen R.J. (2009). Conditional knockdown of hMRS2 results in loss of mitochondrial Mg(2+) uptake and cell death. J. Cell. Mol. Med..

[129] Merolle L., Sponder G., Sargenti A., Mastrototaro L., Cappadone C., Farruggia G., Procopio A., Malucelli E., Parisse P., Gianoncelli A. (2018). Overexpression of the mitochondrial Mg channel MRS2 increases total cellular Mg concentration and influences sensitivity to apoptosis. Metallomics.

[130] De Baaij J.H., Arjona F.J., van den Brand M., Lavrijsen M., Lameris A.L., Bindels R.J., Hoenderop J.G. (2016). Identification of SLC41A3 as a novel player in magnesium homeostasis. Sci. Rep..

[131] Goytain A., Quamme G.A. (2005). Identification and characterization of a novel mammalian Mg^2+^ transporter with channel-like properties. BMC Genom..

[132] Pearson P.J., Evora P.R., Seccombe J.F., Schaff H.V. (1998). Hypomagnesemia inhibits nitric oxide release from coronary endothelium: Protective role of magnesium infusion after cardiac operations. Ann. Thorac. Surg..

[133] Tamura M., Kanno M., Kai T. (2001). Destabilization of neutrophil NADPH oxidase by ATP and other trinucleotides and its prevention by Mg^2+^. Biochim. Biophys. Acta.

[134] Weglicki W.B., Phillips T.M., Freedman A.M., Cassidy M.M., Dickens B.F. (1992). Magnesium-deficiency elevates circulating levels of inflammatory cytokines and endothelin. Mol. Cell. Biochem..

[135] Bussiere F.I., Gueux E., Rock E., Mazur A., Rayssiguier Y. (2002). Protective effect of calcium deficiency on the inflammatory response in magnesium-deficient rats. Eur. J. Nutr..

[136] Maier J.A.M., Malpuech-Brugère C., Zimowska W., Rayssiguier Y., Mazur A. (2004). Low magnesium promotes endothelial cell dysfunction: Implications for atherosclerosis, inflammation and thrombosis. Biochim. Biophys. Acta.

[137] Song Y., Li T.Y., van Dam R.M., Manson J.E., Hu F.B. (2007). Magnesium intake and plasma concentrations of markers of systemic inflammation and endothelial dysfunction in women. Am. J. Clin. Nutr..

[138] Tejero-Taldo M.I., Kramer J.H., Mak Iu T., Komarov A.M., Weglicki W.B. (2006). The nerve-heart connection in the pro-oxidant response to Mg-deficiency. Heart Fail. Rev..

[139] Nielsen F.H. (2010). Magnesium, inflammation, and obesity in chronic disease. Nutr. Rev..

[140] Almoznino-Sarafian D., Berman S., Mor A., Shteinshnaider M., Gorelik O., Tzur I., Alon I., Modai D., Cohen N. (2007). Magnesium and C-reactive protein in heart failure: An anti-inflammatory effect of magnesium administration?. Eur. J. Nutr..

[141] Giacconi R., Muti E., Malavolta M., Cipriano C., Costarelli L., Bernardini G., Gasparini N., Mariani E., Saba V., Boccoli G. (2007). The +838 C/G MT2A polymorphism, metals, and the inflammatory/immune response in carotid artery stenosis in elderly people. Mol. Med..

[142] Blache D., Devaux S., Joubert O., Loreau N., Schneider M., Durand P., Prost M., Gaume V., Adrian M., Laurant P. (2006). Long-term moderate magnesium-deficient diet shows relationships between blood pressure, inflammation and oxidant stress defense in aging rats. Free Radic. Biol. Med..

[143] Adrian M., Chanut E., Laurant P., Gaume V., Berthelot A. (2008). A long-term moderate magnesium-deficient diet aggravates cardiovascular risks associated with aging and increases mortality in rats. J. Hypertens..

[144] Castiglioni S., Cazzaniga A., Maier J.A. (2014). Potential interplay between NFκB and PPARγ in human dermal microvascular endothelial cells cultured in low magnesium. Magnes. Res..

[145] Altura B.M., Shah N.C., Shah G.J., Zhang A., Li W., Zheng T., Perez-Albela J.L., Altura B.T. (2014). Short-term Mg deficiency upregulates protein kinase C isoforms in cardiovascular tissues and cells; relation to NF-kB, cytokines, ceramide salvage sphingolipid pathway and PKC-zeta: Hypothesis and review. Int. J. Clin. Exp. Med..

[146] Chacko S.A., Song Y., Nathan L., Tinker L., de Boer I.H., Tylavsky F., Wallace R., Liu S. (2010). Relations of dietary magnesium intake to biomarkers of inflammation and endothelial dysfunction in an ethnically diverse cohort of postmenopausal women. Diabetes Care.

[147] Weglicki W.B., Mak I.T., Stafford R.E., Dickens B.F., Cassidy M.M., Phillips T.M. (1994). Neurogenic peptides and the cardiomyopathy of magnesium-deficiency: Effects of substance P-receptor inhibition. Mol. Cell. Biochem..

[148] Rude R.K., Gruber H.E., Norton H.J., Wei L.Y., Frausto A., Kilburn J. (2006). Reduction of dietary magnesium by only 50% in the rat disrupts bone and mineral metabolism. Osteoporos. Int..

[149] Weglicki W.B., Mak I.T., Phillips T.M. (1994). Blockade of cardiac inflammation in Mg^2+^ deficiency by substance P receptor inhibition. Circ. Res..

[150] Zghoul N., Alam-Eldin N., Mak I.T., Silver B., Weglicki W.B. (2018). Hypomagnesemia in diabetes patients: Comparison of serum and intracellular measurement of responses to magnesium supplementation and its role in inflammation. Diabetes Metab. Syndr. Obes..

[151] Mak I.T., Kramer J.H., Weglicki W.B. (2003). Suppression of neutrophil and endothelial activation by substance P receptor blockade in the Mg-deficient rat. Magnes. Res..

[152] Guerrero-Romero F., Rodríguez-Morán M. (2006). Hypomagnesemia, oxidative stress, inflammation, and metabolic syndrome. Diabetes Metab. Res. Rev..

[153] Ahokas R.A., Sun Y., Bhattacharya S.K., Gerling I.C., Weber K.T. (2005). Aldosteronism and a proinflammatory vascular phenotype: Role of Mg^2+^, Ca^2+^, and H_2_O_2_ in peripheral blood mononuclear cells. Circulation.

[154] Malpuech-Brugère C., Rock E., Astier C., Nowacki W., Mazur A., Rayssiguier Y. (1998). Exacerbated immune stress response during experimental magnesium deficiency results from abnormal cell calcium homeostasis. Life Sci..

[155] Li F.Y., Chaigne-Delalande B., Kanellopoulou C., Davis J.C., Matthews H.F., Douek D.C., Cohen J.I., Uzel G., Su H.C., Lenardo M.J. (2011). Second messenger role for Mg^2+^ revealed by human T-cell immunodeficiency. Nature.

[156] Mazur A., Maier J.A., Rock E., Gueux E., Nowacki W., Rayssiguier Y. (2007). Magnesium and the inflammatory response: Potential physiopathological implications. Arch. Biochem. Biophys..

[157] Zierler S., Sumoza-Toledo A., Suzuki S., Dúill F.Ó., Ryazanova L.V., Penner R., Ryazanov A.G., Fleig A. (2016). TRPM7 kinase activity regulates murine mast cell degranulation. J. Physiol..

[158] Libako P., Nowacki W., Rock E., Rayssiguier Y., Mazur A. (2010). Phagocyte priming by low magnesium status: Input to the enhanced inflammatory and oxidative stress responses. Magnes. Res..

[159] Ferrè S., Baldoli E., Leidi M., Maier J.A.M. (2010). Magnesium deficiency promotes a pro-atherogenic phenotype in cultured human endothelial cells via activation of NFkB. Biochim. Biophys. Acta.

[160] Bernardini D., Nasulewic A., Mazur A., Maier J.A. (2005). Magnesium and microvascular endothelial cells: A role in inflammation and angiogenesis. Front. Biosci..

[161] Rochelson B., Dowling O., Schwartz N., Metz C.N. (2007). Magnesium sulfate suppresses inflammatory responses by human umbilical vein endothelial cells (HuVECs) through the NFkappaB pathway. J. Reprod. Immunol..

[162] Kohli P., Levy B.D. (2009). Resolvins and protectins: Mediating solutions to inflammation. Br. J. Pharmacol..

[163] Bussière F.I., Gueux E., Rock E., Girardeau J.P., Tridon A., Mazur A., Rayssiguier Y. (2002). Increased phagocytosis and production of reactive oxygen species by neutrophils during magnesium deficiency in rats and inhibition by high magnesium concentration. Br. J. Nutr..

[164] Bussière F.I., Mazur A., Fauquert J.L., Labbe A., Rayssiguier Y., Tridon A. (2002). High magnesium concentration in vitro decreases human leukocyte activation. Magnes. Res..

[165] Montell C. (2003). Mg^2+^ homeostasis: The Mg2+nificent TRPM chanzymes. Curr. Biol..

[166] Bates-Withers C., Sah R., Clapham D.E., Islam M.S. (2011). TRPM7, the Mg^2+^ inhibited channel and kinase. Transient Receptor Potential Channels.

[167] Schlingmann K.P., Gudermann T. (2005). A critical role of TRPM channel-kinase for human magnesium transport. J. Physiol..

[168] Yogi A., Callera G.E., Antunes T.T., Tostes R.C., Touyz R.M. (2011). Transient receptor potential melastatin 7 (TRPM7) cation channels, magnesium and the vascular system in hypertension. Circ. J..

[169] Chokshi R., Matsushita M., Kozak J.A. (2012). Detailed examination of Mg^2+^ and pH sensitivity of human TRPM7 channels. Am. J. Physiol. Cell Physiol..

[170] Kozak J.A., Matsushita M., Nairn A.C., Cahalan M.D. (2005). Charge screening by internal pH and polyvalent cations as a mechanism for activation, inhibition, and rundown of TRPM7/MIC channels. J. Gen. Physiol..

[171] Rios F.J., Zou Z.-G., Harvey A.P., Harvey K.Y., Nosalski R., Anyfanti P., Camargo L.L., Lacchini S., Ryazanov A.G., Ryazanova L. (2019). Chanzyme TRPM7 protects against cardiovascular inflammation and fibrosis. Cardiovasc. Res..

[172] Schilling T., Miralles F., Eder C. (2014). TRPM7 regulates proliferation and polarisation of macrophages. J. Cell Sci..

[173] Li F.Y., Lenardo M.J., Chaigne-Delalande B. (2011). Loss of MAGT1 abrogates the Mg^2+^ flux required for T cell signaling and leads to a novel human primary immunodeficiency. Magnes. Res..

[174] Matsuda-Lennikov M., Biancalana M., Zou J., Ravell J.C., Zheng L., Kanellopoulou C., Jiang P., Notarangelo G., Jing H., Masutani E. (2019). Magnesium transporter 1 (MAGT1) deficiency causes selective defects in N-linked glycosylation and expression of immune-response genes. J. Biol. Chem..

[175] Kostov K., Halacheva L. (2018). Role of magnesium deficiency in promoting atherosclerosis, endothelial dysfunction, and arterial stiffening as risk factors for hypertension. Int. J. Mol. Sci..

[176] Sanui H., Rubin A.H. (1978). Membrane bound and cellular cationic changes associated with insulin stimulation of cultured cells. J. Cell. Physiol..

[177] Jamilian M., Samimi M., Faraneh A.E., Aghadavod E., Shahrzad H.D., Chamani M., Mafi A., Asemi Z. (2017). Magnesium supplementation affects gene expression related to insulin and lipid in patients with gestational diabetes. Magnes. Res..

[178] Solaimani H., Soltani N., MaleKzadeh K., Sohrabipour S., Zhang N., Nasri S., Wang Q. (2014). Modulation of GLUT4 expression by oral administration of Mg^2+^ to control sugar levels in STZ-induced diabetic rats. Can. J. Physiol. Pharmacol..

[179] Van Laecke S., Caluwe R., Huybrechts I., Nagler E.V., Vanholder R., Peeters P., Van Vlem B., Van Biesen W. (2017). Effect of magnesium supplements on insulin secretion after kidney transplantation: A randomized controlled trial. Ann. Transplant..

[180] Rodríguez-Morán M., Guerrero-Romero F. (2011). Insulin secretion is decreased in non-diabetic individuals with hypomagnesaemia. Diabetes Metab. Res. Rev..

[181] Wright E., Scism-Bacon J.L., Glass L.C. (2006). Oxidative stress in type 2 diabetes: The role of fasting and postprandial glycaemia. Int. J. Clin. Pract..

[182] Cunha A.R., Umbelino B., Correia M.L., Neves M.F. (2012). Magnesium and vascular changes in hypertension. Int. J. Hypertens..

[183] Kamran M., Kharazmi F., Malekzadeh K., Talebi A., Khosravi F., Soltani N. (2019). Effect of long-term administration of oral magnesium sulfate and insulin to reduce streptozotocin-induced hyperglycemia in rats: The role of Akt2 and IRS1 gene expressions. Biol. Trace Elem. Res..

[184] Barbagallo M., Dominguez L.J., Tagliamonte M.R., Resnick L.M., Paolisso G. (1999). Effects of vitamin E and glutathione on glucose metabolism: Role of magnesium. Hypertension.

[185] Garfinkel L., Garfinkel D. (1985). Magnesium regulation of the glycolytic pathway and the enzymes involved. Magnesium.

[186] Denton R.M., Randle P.J., Bridges B.J., Cooper R.H., Kerbey A.L., Pask H.T., Severson D.L., Stansbie D., Whitehouse S. (1975). Regulation of mammalian pyruvate dehydrogenase. Mol. Cell. Biochem..

[187] Thomas A.P., Diggle T.A., Denton R.M. (1986). Sensitivity of pyruvate dehydrogenase phosphate phosphatase to magnesium ions. Similar effects of spermine and insulin. Biochem. J..

[188] Mastrototaro L., Tietjen U., Sponder G., Vormann J., Aschenbach J.R., Kolisek M. (2015). Insulin modulates the Na^+^/Mg^2+^ exchanger SLC41A1 and influences Mg^2+^ efflux from intracellular stores in transgenic HEK293 cells. J. Nutr..

[189] Kolisek M., Launay P., Beck A., Sponder G., Serafini N., Brenkus M., Froschauer E.M., Martens H., Fleig A., Schweigel M. (2008). SLC41A1 is a novel mammalian Mg^2+^ carrier. J. Biol. Chem..

[190] Abbott L.G., Rude R.K. (1993). Clinical manifestations of magnesium deficiency. Miner. Electrolyte Metab..

[191] Shafiq A., Goyal A., Jones P.G., Sahil S., Hoffman M., Qintar M., Buchanan D.M., Kosiborod M., Arnold S.V. (2017). Serum magnesium levels and in-hospital mortality in acute myocardial infarction. J. Am. Coll. Cardiol..

[192] Woods K.L., Fletcher S., Roffe C., Haider Y. (1992). Intravenous magnesium sulphate in suspected acute myocardial infarction: Results of the second Leicester Intravenous Magnesium Intervention Trial (LIMIT-2). Lancet.

[193] Woods K.L., Fletcher S. (1994). Long-term outcome after intravenous magnesium sulphate in suspected acute myocardial infarction: The second Leicester Intravenous Magnesium Intervention Trial (LIMIT-2). Lancet.

[194] Ince C., Schulman S.P., Quigley J.F., Berger R.D., Kolasa M., Ferguson R., Silver B., Haigney M.C. (2001). Usefulness of magnesium sulfate in stabilizing cardiac repolarization in heart failure secondary to ischemic cardiomyopathy. Am. J. Cardiol..

[195] Bashir Y., Sneddon J.F., Staunton H.A., Haywood G.A., Simpson I.A., McKenna W.J., Camm A.J. (1993). Effects of long-term oral magnesium chloride replacement in congestive heart failure secondary to coronary artery disease. Am. J. Cardiol..

[196] Gottlieb S.S., Fisher M.L., Pressel M.D., Patten R.D., Weinberg M., Greenberg N. (1993). Effects of intravenous magnesium sulfate on arrhythmias in patients with congestive heart failure. Am. Heart J..

[197] Stepura O.B., Martynow A.I. (2009). Magnesium orotate in severe congestive heart failure (MACH). Int. J. Cardiol..

[198] Terzi A., Furlan G., Chiavacci P., Dal Corso B., Luzzani A., Dalla Volta S. (1996). Prevention of atrial tachyarrhythmias after non-cardiac thoracic surgery by infusion of magnesium sulfate. Thorac. Cardiovasc. Surg..

[199] He D., Aggarwal N., Zurakowski D., Jonas R.A., Berul C.I., Hanumanthaiah S., Moak J.P. (2018). Lower risk of postoperative arrhythmias in congenital heart surgery following intraoperative administration of magnesium. J. Thorac. Cardiovasc. Surg..

[200] Tzivoni D., Banai S., Schuger C., Benhorin J., Keren A., Gottlieb S., Stern S. (1988). Treatment of torsade de pointes with magnesium sulfate. Circulation.

[201] Baker W.L. (2017). Treating arrhythmias with adjunctive magnesium: Identifying future research directions. Eur. Heart J. Cardiovasc. Pharmacother..

[202] Ho K.M. (2008). Intravenous magnesium for cardiac arrhythmias: Jack of all trades. Magnes. Res..

[203] Ho K.M., Sheridan D.J., Paterson T. (2007). Use of intravenous magnesium to treat acute onset atrial fibrillation: A meta-analysis. Heart.

[204] Lomivorotov V.V., Efremov S.M., Karaskov A.M. (2018). Pharmacokinetics of magnesium in cardiac surgery: Implications for prophylaxis against atrial fibrillation. J. Cardiothorac. Vasc. Anesth..

[205] Shiga T., Wajima Z., Inoue T., Ogawa R. (2004). Magnesium prophylaxis for arrhythmias after cardiac surgery: A meta-analysis of randomized controlled trials. Am. J. Med..

[206] Biesenbach P., Mårtensson J., Lucchetta L., Bangia R., Fairley J., Jansen I., Matalanis G., Bellomo R. (2018). Pharmacokinetics of magnesium bolus therapy in cardiothoracic surgery. J. Cardiothorac. Vasc. Anesth..

[207] Davey M.J., Teubner D. (2005). A randomized controlled trial of magnesium sulfate, in addition to usual care, for rate control in atrial fibrillation. Ann. Emerg. Med..

[208] Zaman A.G., Alamgir F., Richens T., Williams R., Rothman M.T., Mills P.G. (1997). The role of signal averaged P wave duration and serum magnesium as a combined predictor of atrial fibrillation after elective coronary artery bypass surgery. Heart.

[209] Kohno H., Koyanagi T., Kasegawa H., Miyazaki M. (2005). Three-day magnesium administration prevents atrial fibrillation after coronary artery bypass grafting. Ann. Thorac. Surg..

[210] Banai S., Tzivoni D. (1993). Drug therapy for torsade de pointes. J. Cardiovasc. Electrophysiol..

[211] Zipes D.P., Camm A.J., Borggrefe M., Buxton A.E., Chaitman B., Fromer M., Gregoratos G., Klein G., Moss A.J., Myerburg R.J. (2006). ACC/AHA/ESC 2006 guidelines for management of patients with ventricular arrhythmias and the prevention of sudden cardiac death--executive summary: A report of the American College of Cardiology/American Heart Association Task Force and the European Society of Cardiology Committee for Practice Guidelines (Writing Committee to Develop Guidelines for Management of Patients with Ventricular Arrhythmias and the Prevention of Sudden Cardiac Death) Developed in collaboration with the European Heart Rhythm Association and the Heart Rhythm Society. Eur. Heart J..

[212] Chinushi M., Sugiura H., Komura S., Hirono T., Izumi D., Tagawa M., Furushima H., Aizawa Y. (2005). Effects of intravenous magnesium in a prolonged QT interval model of polymorphic ventricular tachycardia focus on transmural ventricular repolarization. Pacing Clin. Electrophysiol..

[213] Bailie D.S., Inoue H., Kaseda S., Ben-David J., Zipes D.P. (1988). Magnesium suppression of early afterdepolarizations and ventricular tachyarrhythmias induced by cesium in dogs. Circulation.

[214] Ford E.S. (1999). Serum magnesium and ischaemic heart disease: Findings from a national sample of US adults. Int. J. Epidemiol..

[215] Freedman A.M., Cassidy M.M., Weglicki W.B. (1991). Magnesium-deficient myocardium demonstrates an increased susceptibility to an in vivo oxidative stress. Magnes. Res..

[216] Shi B., Heavner J.E., Boylan L.M., Wang M.J., Spallholz J.E. (1995). Dietary magnesium deficiency increases Giα levels in the rat heart after myocardial infarction. Cardiovasc. Res..

[217] Morton B.C., Nair R.C., Smith F.M., McKibbon T.G., Poznanski W.J. (1984). Magnesium therapy in acute myocardial infarction--a double-blind study. Magnesium.

[218] Rasmussen H.S., McNair P., Norregard P., Backer V., Lindeneg O., Balslev S. (1986). Intravenous magnesium in acute myocardial infarction. Lancet.

[219] Smith L.F., Heagerty A.M., Bing R.F., Barnett D.B. (1986). Intravenous infusion of magnesium sulphate after acute myocardial infarction: Effects on arrhythmias and mortality. Int. J. Cardiol..

[220] Abraham A.S., Rosenmann D., Kramer M., Balkin J., Zion M.M., Farbstien H., Eylath U. (1987). Magnesium in the prevention of lethal arrhythmias in acute myocardial infarction. Arch. Intern. Med..

[221] Shechter M., Hod H., Rabinowitz B., Boyko V., Chouraqui P. (2003). Long-term outcome of intravenous magnesium therapy in thrombolysis-ineligible acute myocardial infarction patients. Cardiology.

[222] Shen A.C., Jennings R.B. (1972). Myocardial calcium and magnesium in acute ischemic injury. Am. J. Pathol..

[223] Vormann J., Fischer G., Classen H.G., Thöni H. (1983). Influence of decreased and increased magnesium supply on the cardiotoxic effects of epinephrine in rats. Arzneimittelforschung.

[224] Parikka H., Toivonen L., Naukkarinen V., Tierala I., Pohjola-Sintonen S., Heikkilä J., Nieminen M.S. (1999). Decreases by magnesium of QT dispersion and ventricular arrhythmias in patients with acute myocardial infarction. Eur. Heart J..

[225] Benz I., Kohlhardt M. (1991). Modulation of single cardiac Na^+^ channels by cytosolic Mg^++^ ions. Eur. Biophys. J..

[226] White R.E., Hartzell H.C. (1988). Effects of intracellular free magnesium on calcium current in isolated cardiac myocytes. Science.

[227] Hartzell H.C., White R.E. (1989). Effects of magnesium on inactivation of the voltage-gated calcium current in cardiac myocytes. J. Gen. Physiol..

[228] Agus Z.S., Kelepouris E., Dukes I., Morad M. (1989). Cytosolic magnesium modulates calcium channel activity in mammalian ventricular cells. Am. J. Physiol..

[229] Wu J.Y., Lipsius S.L. (1990). Effects of extracellular Mg^2+^ on T- and L-type Ca^2+^ currents in single atrial myocytes. Am. J. Physiol..

[230] Wei S.K., Quigley J.F., Hanlon S.U., O’Rourke B., Haigney M.C. (2002). Cytosolic free magnesium modulates Na/Ca exchange currents in pig myocytes. Cardiovasc. Res..

[231] Wei S.K., Hanlon S.U., Haigney M.C. (2002). Beta-adrenergic stimulation of pig myocytes with decreased cytosolic free magnesium prolongs the action potential and enhances triggered activity. J. Cardiovasc. Electrophysiol..

[232] Duchatelle-Gourdon I., Hartzell H.C., Lagrutta A.A. (1989). Modulation of the delayed rectifier potassium current in frog cardiomyocytes by beta-adrenergic agonists and magnesium. J. Physiol..

[233] Duchatelle-Gourdon I., Lagrutta A.A., Hartzell H.C. (1991). Effects of Mg^2+^ on basal and beta-adrenergic-stimulated delayed rectifier potassium current in frog atrial myocytes. J. Physiol..

[234] Ishihara K., Mitsuiye T., Noma A., Takano M. (1989). The Mg^2+^ block and intrinsic gating underlying inward rectification of the K^+^ current in guinea-pig cardiac myocytes. J. Physiol..

[235] Hartzell H.C., Duchatelle-Gourdon I. (1993). Regulation of the cardiac delayed rectifier K current by neurotransmitters and magnesium. Cardiovasc. Drugs Ther..

[236] Hirahara K., Matsubayashi T., Matsuura H., Ehara T. (1998). Intracellular Mg^2+^ depletion depresses the delayed rectifier K^+^ current in guinea pig ventricular myocytes. Jpn. J. Physiol..

[237] Findlay I. (1987). ATP-sensitive K^+^ channels in rat ventricular myocytes are blocked and inactivated by internal divalent cations. Pflug. Arch..

[238] Sharma P., Chung C., Vizcaychipi M. (2014). Magnesium: The neglected electrolyte? A clinical review. Pharmacol. Pharm..

[239] Dunnett J., Nayler W.G. (1978). Calcium efflux from cardiac sarcoplasmic reticulum: Effects of calcium and magnesium. J. Mol. Cell. Cardiol..

[240] Laver D.R., Baynes T.M., Dulhunty A.F. (1997). Magnesium inhibition of ryanodine-receptor calcium channels: Evidence for two independent mechanisms. J. Membr. Biol..

[241] Laver D.R., Honen B.N. (2008). Luminal Mg^2+^, a key factor controlling RYR2-mediated Ca^2+^ release: Cytoplasmic and luminal regulation modeled in a tetrameric channel. J. Gen. Physiol..

[242] Bara M., Guiet-Bara A., Durlach J. (1993). Regulation of sodium and potassium pathways by magnesium in cell membranes. Magnes. Res..

[243] Ishihara K., Sarai N., Asakura K., Noma A., Matsuoka S. (2009). Role of Mg^2+^ block of the inward rectifier K^+^ current in cardiac repolarization reserve: A quantitative simulation. J. Mol. Cell. Cardiol..

[244] Choudhary G., Dudley S.C. (2002). Heart failure, oxidative stress, and ion channel modulation. Congest. Heart Fail..

[245] Ayoub K.F., Pothineni N.V.K., Rutland J., Ding Z., Mehta J.L. (2017). Immunity, inflammation, and oxidative stress in heart failure: Emerging molecular targets. Cardiovasc. Drugs Ther..

[246] Dudley S.C., Hoch N.E., McCann L.A., Honeycutt C., Diamandopoulos L., Fukai T., Harrison D.G., Dikalov S.I., Langberg J. (2005). Atrial fibrillation increases production of superoxide by the left atrium and left atrial appendage: Role of the NADPH and xanthine oxidases. Circulation.

[247] Hanna I.R., Heeke B., Bush H., Brosius L., King-Hageman D., Dudley S.C., Beshai J.F., Langberg J.J. (2006). Lipid-lowering drug use is associated with reduced prevalence of atrial fibrillation in patients with left ventricular systolic dysfunction. Heart Rhythm.

[248] Gao G., Dudley S.C. (2009). Redox regulation, NF-kB, and atrial fibrillation. Antioxid. Redox Signal..

[249] Chung M.K., Martin D.O., Sprecher D., Wazni O., Kanderian A., Carnes C.A., Bauer J.A., Tchou P.J., Niebauer M.J., Natale A. (2001). C-reactive protein elevation in patients with atrial arrhythmias: Inflammatory mechanisms and persistence of atrial fibrillation. Circulation.

[250] Aviles R.J., Martin D.O., Apperson-Hansen C., Houghtaling P.L., Rautaharju P., Kronmal R.A., Tracy R.P., Van Wagoner D.R., Psaty B.M., Lauer M.S. (2003). Inflammation as a risk factor for atrial fibrillation. Circulation.

[251] Liu M., Liu H., Dudley S.C. (2010). Reactive oxygen species originating from mitochondria regulate the cardiac sodium channel. Circ. Res..

[252] Jeong E.M., Liu M., Sturdy M., Gao G., Sovari A.A., Dudley J. (2011). Metabolic stress, reactive oxygen species, and arrhythmia. J. Mol. Cell. Cardiol..

[253] Liu M., Shi G., Yang K.C., Gu L., Kanthasamy A.G., Anantharam V., Dudley J. (2017). Role of protein kinase C in metabolic regulation of the cardiac Na^+^ channel. Heart Rhythm.

[254] Nakajima T., Davies S.S., Matafonova E., Potet F., Amarnath V., Tallman K.A., Serwa R.A., Porter N.A., Balser J.R., Kupershmidt S. (2010). Selective g-ketoaldehyde scavengers protect Na_v_1.5 from oxidant-induced inactivation. J. Mol. Cell. Cardiol..

[255] Zhao Z., Xie Y., Wen H., Xiao D., Allen C., Fefelova N., Dun W., Boyden P.A., Qu Z., Xie L.H. (2012). Role of the transient outward potassium current in the genesis of early afterdepolarizations in cardiac cells. Cardiovasc. Res..

[256] Coetzee W.A., Opie L.H. (1992). Effects of oxygen free radicals on isolated cardiac myocytes from guinea-pig ventricle: Electrophysiological studies. J. Mol. Cell. Cardiol..

[257] Fearon I.M., Palmer A.C.V., Balmforth A.J., Ball S.G., Varadi G., Peers C. (1999). Modulation of recombinant human cardiac L-type Ca^2+^ channel a_1C_ subunits by redox agents and hypoxia. J. Physiol..

[258] Hool L.C., Di Maria C.A., Viola H.M., Arthur P.G. (2005). Role of NAD(P)H oxidase in the regulation of cardiac L-type Ca^2+^ channel function during acute hypoxia. Cardiovasc. Res..

[259] DeSantiago J., Bare D.J., Varma D., Solaro R.J., Arora R., Banach K. (2018). Loss of p21-activated kinase 1 (Pak1) promotes atrial arrhythmic activity. Heart Rhythm.

[260] Cherednichenko G., Zima A.V., Feng W., Schaefer S., Blatter L.A., Pessah I.N. (2004). NADH oxidase activity of rat cardiac sarcoplasmic reticulum regulates calcium-induced calcium release. Circ. Res..

[261] Gao L., Li Y., Schultz H.D., Wang W.Z., Wang W., Finch M., Smith L.M., Zucker I.H. (2010). Downregulated K_v_4.3 expression in the RVLM as a potential mechanism for sympathoexcitation in rats with chronic heart failure. Am. J. Physiol. Heart Circ. Physiol..

[262] Dong D., Liu Y., Zhou Y., Song W., Wang H., Yang B. (2004). Decreases of voltage-dependent K^+^ currents densities in ventricular myocytes of guinea pigs by chronic oxidant stress. Acta Pharmacol. Sin..

[263] Sunagawa T., Shimizu T., Matsumoto A., Tagashira M., Kanda T., Shirasawa T., Nakaya H. (2014). Cardiac electrophysiological alterations in heart/muscle-specific manganese-superoxide dismutase-deficient mice: Prevention by a dietary antioxidant polyphenol. BioMed Res. Int..

[264] Yalta T., Yalta K. (2018). Systemic inflammation and arrhythmogenesis: A review of mechanistic and clinical perspectives. Angiology.

[265] Korantzopoulos P., Letsas K.P., Tse G., Fragakis N., Goudis C.A., Liu T. (2018). Inflammation and atrial fibrillation: A comprehensive review. J. Arrhythmia.

[266] Song Y., He K., Levitan E.B., Manson J.E., Liu S. (2006). Effects of oral magnesium supplementation on glycaemic control in type 2 diabetes: A meta-analysis of randomized double-blind controlled trials. Diabet. Med..

[267] Bertinato J., Xiao C.W., Ratnayake W.M., Fernandez L., Lavergne C., Wood C., Swist E. (2015). Lower serum magnesium concentration is associated with diabetes, insulin resistance, and obesity in South Asian and white Canadian women but not men. Food Nutr. Res..

[268] Schutten J.C., Gomes-Neto A.W., Navis G., Gansevoort R.T., Dullaart R.P.F., Kootstra-Ros J.E., Danel R.M., Goorman F., Gans R.O.B., de Borst M.H. (2019). Lower plasma magnesium, measured by nuclear magnetic resonance spectroscopy, is associated with increased risk of developing type 2 diabetes mellitus in women: Results from a Dutch prospective cohort study. J. Clin. Med..

[269] Simental-Mendía L.E., Sahebkar A., Rodríguez-Morán M., Guerrero-Romero F. (2016). A systematic review and meta-analysis of randomized controlled trials on the effects of magnesium supplementation on insulin sensitivity and glucose control. Pharmacol. Res..

[270] Guerrero-Romero F., Simental-Mendía L.E., Hernández-Ronquillo G., Rodriguez-Morán M. (2015). Oral magnesium supplementation improves glycaemic status in subjects with prediabetes and hypomagnesaemia: A double-blind placebo-controlled randomized trial. Diabetes Metab..

[271] Hatzistavri L.S., Sarafidis P.A., Georgianos P.I., Tziolas I.M., Aroditis C.P., Zebekakis P.E., Pikilidou M.I., Lasaridis A.N. (2009). Oral magnesium supplementation reduces ambulatory blood pressure in patients with mild hypertension. Am. J. Hypertens..

[272] Shechter M., Merz C.N., Rude R.K., Paul Labrador M.J., Meisel S.R., Shah P.K., Kaul S. (2000). Low intracellular magnesium levels promote platelet-dependent thrombosis in patients with coronary artery disease. Am. Heart J..

[273] Rayssiguier Y., Noé L., Etienne J., Gueux E., Cardot P., Mazur A. (1991). Effect of magnesium deficiency on post-heparin lipase activity and tissue lipoprotein lipase in the rat. Lipids.

[274] Bussière L., Mazur A., Gueux E., Nowacki W., Rayssiguier Y. (1995). Triglyceride-rich lipoproteins from magnesium-deficient rats are more susceptible to oxidation by cells and promote proliferation of cultured vascular smooth muscle cells. Magnes. Res..

[275] Martínez-Revelles S., García-Redondo A.B., Avendaño M.S., Varona S., Palao T., Orriols M., Roque F.R., Fortuño A., Touyz R.M., Martínez-González J. (2017). Lysyl oxidase induces vascular oxidative stress and contributes to arterial stiffness and abnormal elastin structure in hypertension: Role of p38MAPK. Antioxid. Redox Signal..

[276] Gotru S.K., Chen W., Kraft P., Becker I.C., Wolf K., Stritt S., Zierler S., Hermanns H.M., Rao D., Perraud A.L. (2018). TRPM7 kinase controls calcium responses in arterial thrombosis and stroke in mice. Arterioscler. Thromb. Vasc. Biol..

[277] Lazard E.M. (1925). A preliminary report on the intravenous use of magnesium sulphate in puerperal eclampsia. Am. J. Obstet. Gynecol..

[278] Gabbe S.G. (1996). Classic pages from the American Journal of obstetrics and Genecology: A preliminary report on the intravenous use of magnesium sulphate in puerperal eclampsia. Am. J. Obstet. Gynecol..

[279] Zuspan F.P. (1966). Treatment of severe preeclampsia and eclampsia. Clin. Obstet. Gynecol..

[280] Altman D., Carroli G., Duley L., Farrell B., Moodley J., Neilson J., Smith D. (2002). Do women with pre-eclampsia, and their babies, benefit from magnesium sulphate? The Magpie Trial: A randomised placebo-controlled trial. Lancet.

[281] Duley L., Gülmezoglu A.M., Henderson-Smart D.J., Chou D. (2010). Magnesium sulphate and other anticonvulsants for women with pre-eclampsia. Cochrane Database Syst. Rev..

[282] Lu J.F., Nightingale C.H. (2000). Magnesium sulfate in eclampsia and pre-eclampsia: Pharmacokinetic principles. Clin. Pharmacokinet..

[283] Okusanya B.O., Oladapo O.T., Long Q., Lumbiganon P., Carroli G., Qureshi G., Duley L., Souza J.P., Gülmezoglu A.M. (2016). Clinical pharmacokinetic properties of magnesium sulphate in women with pre-eclampsia and eclampsia. BJOG.

[284] Lox C.D., Dorsett M.M., Hampton R.M. (1983). Observations on clotting activity during pre-eclampsia. Clin. Exp. Hypertens. B.

[285] Ariza A.C., Bobadilla N., Díaz L., Avila E., Larrea F., Halhali A. (2009). Placental gene expression of calcitonin gene-related peptide and nitric oxide synthases in preeclampsia: Effects of magnesium sulfate. Magnes. Res..

[286] Fei X., Hongxiang Z., Qi C., Daozhen C. (2012). Maternal plasma levels of endothelial dysfunction mediators including AM, CGRP, sICAM-1 and tHcy in pre-eclampsia. Adv. Clin. Exp. Med..

[287] Chiuve S.E., Korngold E.C., Januzzi J.L., Gantzer M.L., Albert C.M. (2011). Plasma and dietary magnesium and risk of sudden cardiac death in women. Am. J. Clin. Nutr..

[288] Zhang W., Iso H., Ohira T., Date C., Tamakoshi A. (2012). Associations of dietary magnesium intake with mortality from cardiovascular disease: The JACC study. Atherosclerosis.

[289] Ye H., Cao P., Zhang X., Lin J., Guo Q., Mao H., Yu X., Yang X. (2018). Serum magnesium and cardiovascular mortality in peritoneal dialysis patients: A 5-year prospective cohort study. Br. J. Nutr..

[290] Xu T., Sun Y., Xu T., Zhang Y. (2013). Magnesium intake and cardiovascular disease mortality: A meta-analysis of prospective cohort studies. Int. J. Cardiol..

[291] Christiansen E.H., Frost L., Andreasen F., Mortensen P., Thomsen P.E., Pedersen A.K. (2000). Dose-related cardiac electrophysiological effects of intravenous magnesium. A double-blind placebo-controlled dose-response study in patients with paroxysmal supraventricular tachycardia. Europace.

[292] Ranade V.V., Somberg J.C. (2001). Bioavailability and pharmacokinetics of magnesium after administration of magnesium salts to humans. Am. J. Ther..

[293] Coudray C., Rambeau M., Feillet-Coudray C., Gueux E., Tressol J.C., Mazur A., Rayssiguier Y. (2005). Study of magnesium bioavailability from ten organic and inorganic Mg salts in Mg-depleted rats using a stable isotope approach. Magnes. Res..

[294] Firoz M., Graber M. (2001). Bioavailability of US commercial magnesium preparations. Magnes. Res..

[295] Baker W.L., Kluger J., White C.M., Dale K.M., Silver B.B., Coleman C.I. (2009). Effect of magnesium L-lactate on blood pressure in patients with an implantable cardioverter defibrillator. Ann. Pharmacother..

[296] Brodsky M.A., Orlov M.V., Capparelli E.V., Allen B.J., Iseri L.T., Ginkel M., Orlov Y.S.K. (1994). Magnesium therapy in new-onset atrial fibrillation. Am. J. Cardiol..

[297] Ingemansson M.P., Smideberg B., Olsson S.B. (2000). Intravenous MgSO_4_ alone and in combination with glucose, insulin and potassium (GIK) prolong the atrial cycle length in chronic atrial fibrillation. Europace.

[298] Ceremuzyński L., Jurgiel R., Kulakowski P., Gebalska J. (1989). Threatening arrhythmias in acute myocardial infarction are prevented by intravenous magnesium sulfate. Am. Heart J..

[299] Winters S.L., Sachs R.G., Curwin J.H. (1997). Nonsustained polymorphous ventricular tachycardia during amiodarone therapy for atrial fibrillation complicating cardiomyopathy. Management with intravenous magnesium sulfate. Chest.

[300] Sarisoy O., Babaoglu K., Tugay S., Barn E., Gokalp A.S. (2007). Efficacy of magnesium sulfate for treatment of ventricular tachycardia in amitriptyline intoxication. Pediatr. Emerg. Care.

[301] Rasmussen H.S., Thomsen P.E. (1989). The electrophysiological effects of intravenous magnesium on human sinus node, atrioventricular node, atrium, and ventricle. Clin. Cardiol..

[302] England M.R., Gordon G., Salem M., Chernow B. (1992). Magnesium administration and dysrhythmias after cardiac surgery. A placebo-controlled, double-blind, randomized trial. JAMA.

[303] Colquhoun I.W., Berg G.A., el-Fiky M., Hurle A., Fell G.S., Wheatley D.J. (1993). Arrhythmia prophylaxis after coronary artery surgery. A randomised controlled trial of intravenous magnesium chloride. Eur. J. Cardiothorac. Surg..

[304] Li S., Tian H. (1997). Oral low-dose magnesium gluconate preventing pregnancy induced hypertension. Chin. J. Obstet. Gynecol..

[305] Manz M., Jung W., Lüderitz B. (1997). Effect of magnesium on sustained ventricular tachycardia. Herz.

[306] Caron M.F., Kluger J., Tsikouris J.P., Ritvo A., Kalus J.S., White C.M. (2003). Effects of intravenous magnesium sulfate on the QT interval in patients receiving ibutilide. Pharmacotherapy.

[307] Kalus J.S., Spencer A.P., Tsikouris J.P., Chung J.O., Kenyon K.W., Ziska M., Kluger J., White C.M. (2003). Impact of prophylactic i.v. magnesium on the efficacy of ibutilide for conversion of atrial fibrillation or flutter. Am. J. Health Syst. Pharm..

[308] Leor J., Kloner R.A. (1995). An experimental model examining the role of magnesium in the therapy of acute myocardial infarction. Am. J. Cardiol..

[309] Barr C.S., Lang C.C., Hanson J., Arnott M., Kennedy N., Struthers A.D. (1995). Effects of adding spironolactone to an angiotensin-converting enzyme inhibitor in chronic congestive heart failure secondary to coronary artery disease. Am. J. Cardiol..

[310] Netzer T., Knauf H., Mutschler E. (1992). Modulation of electrolyte excretion by potassium retaining diuretics. Eur. Heart J..

[311] Schwinger R.H., Antoni D.H. (1992). Triamterene may preserve lymphocyte magnesium and potassium in patients with congestive heart failure. Magnes. Res..

[312] Magnesium—Fact Sheet for Health Professionals. https://ods.od.nih.gov/factsheets/Magnesium-HealthProfessional/.

[313] Fang X., Wang K., Han D., He X., Wei J., Zhao L., Imam M.U., Ping Z., Li Y., Xu Y. (2016). Dietary magnesium intake and the risk of cardiovascular disease, type 2 diabetes, and all-cause mortality: A dose-response meta-analysis of prospective cohort studies. BMC Med..

[314] Best Diets for Healthy Eating. https://health.usnews.com/best-diet/best-healthy-eating-diets.

[315] Your Guide to Lowering Your Blood Pressure with DASH. https://www.nhlbi.nih.gov/files/docs/public/heart/new_dash.pdf.

[316] Oesterle A., Weber B., Tung R., Choudhry N.K., Singh J.P., Upadhyay G.A. (2018). Preventing postoperative atrial fibrillation after noncardiac surgery: A meta-analysis. Am. J. Med..

[317] Haigney M.C., Berger R., Schulman S., Gerstenblith G., Tunin C., Silver B., Silverman H.S., Tomaselli G., Calkins H. (1997). Tissue magnesium levels and the arrhythmic substrate in humans. J. Cardiovasc. Electrophysiol..

[318] Farouque H.M., Sanders P., Young G.D. (2000). Intravenous magnesium sulfate for acute termination of sustained monomorphic ventricular tachycardia associated with coronary artery disease. Am. J. Cardiol..

[319] Baker W.L., Kluger J., Coleman C.I., White C.M. (2015). Impact of magnesium L-lactate on occurrence of ventricular arrhythmias in patients with implantable cardioverter defibrillators: A randomized, placebo-controlled trial. Open Cardiovasc. Med. J..

[320] Feldstedt M., Boesgaard S., Bouchelouche P., Svenningsen A., Brooks L., Lech Y., Aldershvile J., Skagen K., Godtfredsen J. (1991). Magnesium substitution in acute ischaemic heart syndromes. Eur. Heart J..

[321] Eichhorn E.J., Tandon P.K., DiBianco R., Timmis G.C., Fenster P.E., Shannon J., Packer M. (1993). Clinical and prognostic significance of serum magnesium concentration in patients with severe chronic congestive heart failure: The PROMISE Study. J. Am. Coll. Cardiol..

[322] Bouida W., Beltaief K., Msolli M.A., Azaiez N., Ben Soltane H., Sekma A., Trabelsi I., Boubaker H., Grissa M.H., Methemem M. (2019). Low-dose Magnesium Sulfate Versus High Dose in the Early Management of Rapid Atrial Fibrillation: Randomized Controlled Double-blind Study (LOMAGHI Study). Acad. Emerg. Med..

[323] Adams N. (2019). Magnesium for atrial fibrillation. Emerg. Med. Australas..

